# Challenges and coping mechanisms among women living with unrepaired obstetric fistula in Ethiopia: A phenomenological study

**DOI:** 10.1371/journal.pone.0275318

**Published:** 2022-09-29

**Authors:** Bekana Fekecha Hurissa, Zewdie Birhanu Koricha, Lelisa Sena Dadi

**Affiliations:** 1 School of Midwifery, Institute of Health, Jimma University, Jimma, Ethiopia; 2 Department of Health, Behavior, and Society, Faculty of Public Health, Jimma University, Jimma, Ethiopia; 3 Department of Epidemiology, Faculty of Public Health, Jimma University, Jimma, Ethiopia; Torrens University Australia, AUSTRALIA

## Abstract

**Introduction:**

Obstetric fistula remains a debilitating complication of childbirth and maternal morbidity in developing countries. Few studies document the challenges and coping mechanisms among women living with obstetric fistula in Ethiopia. Therefore, this study aimed to explore the challenges and coping mechanisms among women with obstetric fistula in Ethiopia.

**Methods:**

A phenomenological study was employed among purposively selected eleven women with obstetric fistula and three key informants at five fistula treatment centers in Ethiopia. An in-depth interview was conducted, audio-recorded, and transcribed into a Microsoft Word document. The transcripts were imported into Atlas. ti version 8.4 for thematic analyses.

**Results:**

Painful social life, consequences of fistula, and coping mechanisms with fistula problems were the main themes in this study. Difficult social life, stigma, discrimination, impaired marital status; psychological, physical, sexual, and reproductive health problems were the major challenges for women with obstetric fistulas. Women with fistulas used coping mechanisms such as—going to spiritual sites and drinking alcohol to cope with their fistula disease; separating themselves from community participation and living alone in the forest to cope with a painful social life; restricting the amount of drinking water and wearing many clothes at a time to cope with wetness and odors, and allowing their husband to marry a new wife to cope with the impaired marital responsibilities.

**Conclusion:**

Women with obstetric fistulas encountered challenges such as a painful social life, impaired marital status, psychological, physical, sexual, and reproductive health problems; and used coping mechanisms with their fistula condition, difficult social life, and impaired marital responsibility that may have an added negative effect on their overall health. Therefore, policymakers need to prioritize the availability and early utilization of obstetric fistula surgery in all settings for all women living with obstetric fistulas to restore their holistic health.

## Introduction

Obstetric fistula is an extreme and debilitating hardship of childbirth and maternal morbidity. Although the most common cause of obstetric fistula is obstructed labor, iatrogenic causes such as obstetric maneuvers, destructive delivery, operative injury at the time of cesarean section with or without hysterectomy, symphysiotomy, and elective abortion procedures are usually in the absence of skilled medical staff assistance may also be responsible for obstetric fistula in low-income countries [1, 2]. Obstetric fistula has devastating effects on women’s lives and remains the prevalent cause of maternal morbidity in the developing world [3, 4]. Women with obstetric fistulas remain chronic patients and experience constant leakage of urine and/or feces causing major physical, psychosocial, and economic trauma [5]. Many women with obstetric fistulas experience varying degrees of social stigma [6]. A global public health report on obstetric fistulas found that women with fistulas who usually have incontinence are equally affected and live a stigmatized life with social, economic, psychological, reproductive, and sexual effects varying in degree from one setting to another [7].

The World Health Organization and a systematic review report show that an estimated 2–3 million women and ladies worldwide live with obstetric fistulas [8, 9]. Globally, an estimated incidence of 50,000–100,000 new women with fistulas are occurring every year [10, 11]. The vast majority of women with obstetric fistulas are in Africa and Asia [8, 9, 11]. The prevalence of obstetric fistula in South Asia was 1.20 per 1,000 women of reproductive age [9]. In sub-Saharan African countries, 30,000–130,000 new women with fistulas have been identified and recorded annually [10]. It is estimated that Ethiopia has the highest number of women of reproductive age who have experienced obstetric fistula symptoms, estimated at 140,500. The lifetime magnitude of occurrence of obstetric fistula in Ethiopia was 7.3 per 1,000 (95% CI: 5.9–8.7) for women aged 15–49 years. This gives a total burden of 142,387 (95% CI: 115,080, 169,694) women with obstetric fistula in Ethiopia [12].

Evidence shows that many women and girls with obstetric fistulas faced many psychological problems such as humiliation (reported by 97.2% of victims); abandonment, stigmatization, and loneliness (reported by 95.8%) [13–15]. Similar studies showed that 85% of women experienced separation from their husbands, and 95.4% reported despair [15, 16]. Women with obstetric fistulas are often afraid and uncertain about their future with sexual partners, marriage, sex, pregnancy, childbirth, and reintegration into the neighborhood community [17–19]. Similarly, other evidence in the developing world shows a divorce rate of 36% to 67%, a stillborn rate of 55.6% to 85%, childlessness of 83.3%, frequent maternal loss of self-esteem, depression, and suicidal thoughts [20]. Urinary inconsistency due to obstetric fistula affects patient self-esteem and quality of life. These can lead to secondary morbidity, disability, infertility, and significant costs for women with obstetric fistula [21]. However, due to more than one factor, women with fistulas nevertheless live in silence with suffering, and repairing their emotional harm has not gotten much emphasis so far [22].

Even though few studies explored the challenges and coping mechanisms used by women with fistulas in Ethiopia, they were not in comprehensive way and did not identify for which challenges the identified coping mechanisms have been used [23–26]. Therefore, to address the existing knowledge gap, this study aimed to explore the challenges and coping mechanisms with fistula problems among women with obstetric fistula in Ethiopia. The findings of the survey will be used as an essential source of information for drafting strategies and programs to educate such women on early access to surgical care, appropriate coping mechanisms, and the detrimental consequences of some of the coping mechanisms being used by them with fistula conditions for assuaging the challenges they have been going through and restoring their dignity.

## Materials and methods

### Study setting and period

The study was conducted from 1 April to 1 August 2019 at five fistula treatment centers in Ethiopia: Jimma University Medical Center, Asella Hospital, Harar, Mettu, and Addis Ababa Hamlin fistula centers. Addis Ababa Hamlin Fistula Treatment Center offers comprehensive care for women who have been experiencing incontinence due to an obstetric fistula. The hospital has a 120-bed capacity with more than 2,000 women treated per year [27, 28]. Jimma University Medical Center has a gynecology and obstetrics department with a fistula unit. It has an estimated 93 women with obstetric fistula annually [29]. Mettu Hamlin fistula center is located in the southwest part of the country. It treats 199 women annually [30]. Harar Hamlin fistula center treats more than 480 women with obstetric fistula annually [31]. Asella Hospital is equipped with 15 fistula beds and treats more than 90 women with obstetric fistula annually [28].

### Study design

A facility-based explorative phenomenological study design was employed. A phenomenological study is more suitable to explore the challenges and coping mechanisms women with fistulas experienced while living with fistulas.

### Study population and eligibility criteria

The study populations were women with obstetric fistula and key informants (a male health officer, the husband of a woman with fistulas, and a female nurse). We included these two groups of participants to get more rich data on the lived experiences of women living with fistulas concerning the challenges they have been experiencing and the measures they have taken to cope with those challenges and with other obstetric fistula sequelae. Key informants were included to supplement, support, and enrich more the information received from women with fistulas. Moreover, the inclusion of the two groups of participants was used for ensuring the trustworthiness of our data which was confirmed through triangulation of both groups’ data. The inclusion criteria we used for key informants were those who had reported having contact and knowing more about women living with obstetric fistulas; healthcare providers who had reported having experience in providing care for women with fistulas for at least six months; and family members those who were living with women with fistula for at least six months, and reported know more about the living experience of such women. Women with fistulas who were unable to continue with the interview process due to severe fistula complications and those in immediate postoperative units were excluded from the study.

### Sample size and sampling technique

In-depth interviews were conducted with purposively selected eleven women with obstetric fistula and three key informants. The purposive sampling technique was used considering the maximum variation sampling technique involving women with obstetric fistula from different facilities with different background features. We identified the participants based on: the background data received from the participants at the initial in-depth interview phase, the information gotten from healthcare providers at each fistula treatment center, and the information reviewed about the background of women with fistulas from their card records. The sample size selected in this study was based on the principle of data saturation. Based on the recent evidence, we based our definition of data saturation as, the unnecessity of further data collection on the basis of the data that has been collected; how much data (usually number of interviews) is needed until nothing new is apparent (informational redundancy), and as to be consistent with the research question(s), the theoretical position and analytic framework adopted [32]. Accordingly, per our research question, method, sampling technique, and homogeneity of study participants, the adequacy of the data was confirmed that data saturation occurs among eleven women with obstetric fistula that is data redundancy started to be seen after ten participants. The number of participants interviewed continued to the eleventh participant, and accordingly, the sample size was decided based on the richness of the information or the saturation of the data reached. Then, three key informants were purposively selected and interviewed to complement the data obtained from women with fistulas.

### Data collection tools and procedures

Two in-depth interview guides were used(one for women with fistula and the other for key informants). The interview guides were first prepared in English, translated into Afan Oromo (the local language), then back-translated, and rechecked by a third person for consistency. Before data collection, the instruments were also pretested among two key informants (one family member and one health care provider) and three women with a fistula at Jimma University Medical Center fistula unit, and based on the pretest data the interview guides were reframed.

One-on-one in-depth interviews were conducted for collecting data from women with obstetric fistula and key informants through face-to-face interactions at the five hospitals. Six data collectors: (three females, three males), including the first author (male), and five field assistants (two females and three males) collected the data. They were all lecturers, had master’s degrees, and had considerable experience in conducting qualitative interviews. The data collectors and assistants were trained for two days to acquaint them with the research instruments and objectives. All interviews were conducted at separate locations/private rooms where others could not listen to the interviews and in the language that the respondents speak. The time and venues for the interviews were pre-arranged and decided by the first author with the support of the nursing head offices or nurse mentors. The interviews lasted from 60 minutes to 90 minutes. The data were audio-recorded with the permission of the study participants, and field notes (hand-written) were taken. Probes were used to obtain detailed information from the respondents whenever necessary. Interviews continued until the data saturation level.

### Trustworthiness

The credibility of this study was ensured using the triangulation strategy between the results of women with obstetric fistula and key informants. Subjectivity was ruled out by using bracketing (the interview guide was developed without being influenced or dependent extensively on literature review evidence). Transferability was ensured by a clear and thick description of the study settings, providing the sample size, sampling techniques, and the thematic table of the study. Dependability was ensured by conducting peer debriefings among data collectors for getting their alternative explanations and similar consensus on emerging codes. Conformability was ensured through bracketing (i.e., the interpretations of the data were derived from the collected data and not based on the researcher’s viewpoints).

### Data analysis

Initially, interviews were transcribed verbatim using the local language (Afan Oromo). Then, per Brislin’s model of translation, we recruited two competent bilingual translators who were familiar with the research, one to translate the transcripts forward from the local language to English and another to translate them back to the original language without having seen the original text [33]. Finally, both versions were compared to check accuracy, clarity, consistency, and equivalence. Moreover, any discrepancies that occurred during the process were negotiated between the two bilingual translators. Transcribed interviews were compared with field notes and proofread while listening to the audio recordings. Then, the transcripts were prepared for analysis. Throughout data analysis and synthesis, transcripts were also checked and rechecked against the translated interpretations. Furthermore, clarity and consistency between the data presented and the findings were ensured by presenting the description of data-driven themes and sub-themes illustrated with participant quotations. In this study, a thematic analysis method was employed for analysis and writing. It used Braun and Crack’s thematic analysis approach [34]. Braun and Clarke’s thematic analysis proposes six steps to analyze qualitative data: (i) familiarisation with the data through repeated reading of the transcripts; (ii) generation of initial codes across the entire data set followed by close coding for comparing data relevant to each code; (iii) searching for potential themes and sub-themes by sorting different codes into candidate themes and sub-themes; (iv) reviewing and refining the themes and sub-themes by reading all the collated extracts for each theme and sub-theme for checking their coherent pattern; (v) defining and naming the themes and sub-themes through the identification of the relevances of what each theme was about (as well as themes overall) and the determination of what aspect of the data each theme captured; and (vi) producing the report. Accordingly, the first author Bekana Fekecha (BF) and co-investigators read and reread the transcriptions to ensure familiarity with the data and to obtain codes for thematic analysis. The team independently performed data-driven open coding on a set of transcripts from each interview category. Then, all authors verified and reached a consensus on a final list of codes. Then, the rest of the primary raw data were coded. Finally, codes were filtered and merged, codes linked to codes, codes categorized and family coded, coded to sub-themes, and then finally super-coded into main themes. Codes, family codes, sub-themes, and themes emerged directly and inductively from the raw data. The coding and ordinary analysis had been carried out using ATLAS. ti version 8.4 software program ATLAS. ti GmbH, Berlin. Finally, the narrative qualitative information was organized, integrated, and presented in tables, with a figure of networks, and interpreted according to themes, subthemes, and quotes.

### Ethical approval and consent to participate

The protocol for the study was reviewed and approved by the Institutional Review Board (IRB) of Jimma University (Ref.No: IRB 000281/2019). All interviewees signed an informed consent form before participation. Assent was received from teenagers less than 18 years of age and consent was obtained from parents or guardians. Written permission was sanctioned to record the verbal exchange. All study procedures followed the relevant guidelines and regulations of the Helsinki Declaration. All necessary measures were taken and ensured: the dignity, autonomy, interest, well-being, and rights of the study participants throughout the study.

## Results

### Socio-demographic characteristics

Fourteen participants, 11 women with obstetric fistula, and three key informants (two health care providers and one family member) participated in this study. Most women with fistula were in the age range from 30 to 34 years. Most of them were unable to read/write, were in a marital union, have been farmers, and were from rural settings (Tables 1 and 2).

**Table 1:** Socio-demographic characteristics of women with obstetric fistula participated in an in-depth interview at Addis Ababa and Oromia Fistula centers, April to August 2019 (n = 11).

Characteristic	Number of women (n = 11)	Percentage (%)
**Age at interview**		
<20	1	9.1
20–24	1	9.1
25–29	2	18.2
30–34	4	36.4
≥35	3	27.2
**Educational status**		
Not educated	9	81.8
Primary school	1	9.1
Secondary school	1	9.1
**Marital status**		
Married	6	54.5
Divorced	3	27.3
Widowed	2	18.2
**Occupation**		
Farmer	7	63.6
Daily laborer	1	9.1
Merchant	1	9.1
Student	2	18.2
**Residence**		
Urban	1	9.1
Rural	10	90.9

**Table 2:** Socio-demographic characteristics of key informants involved in an in-depth interview at Addis Ababa and Oromia Fistula centers, April to August 2019 (n = 3).

Characteristic	Number of participants (n = 3)	Percentage (%)
**Types of supplementary informants**		
Family member (Male)	1	33.3
^a^HCPs (1 Male & 1 Female)	2	66.7
**Age**		
30–40	1	33.3
>40	2	66.7
**Educational status**		
Primary school	1	33.3
College/University	2	66.7
**Occupation**		
Farmer	1	33.3
Non-government employee	2	66.7
**Residence**		
Urban	2	66.7
Rural	1	33.3

^a^ health care provider.

This study analyzed fourteen primary documents, which were coded to 76 codes, three major themes, and fourteen subthemes (Table 3).

**Table 3:** Themes of challenges and coping mechanisms among women with obstetric fistula in Ethiopia, April to August 2019 (n = 14).

S. No	Main theme	Sub-themes
1.	Painful social life	Discrimination due to fistula Enacted stigma Perceived stigma Limitations on religious practices Limitations on social life participation
2.	Consequences of fistula	Psychological health problems Physical health problems Sexual and reproductive health problems Limitations to daily activities Impaired marital status
3.	Coping mechanisms with obstetric fistula problems	Coping with fistula condition/disease as all Coping with difficult social life Coping with wetness and odor Coping with impaired marital responsibility

### Painful social life

Participants reported numerous challenges of living with an obstetric fistula. The subthemes under this theme were discrimination due to fistula, enacted stigma, perceived stigma, limitations on religious practices, and limitations on social life participation.

#### Discrimination due to fistula

Many participants reported that they had encountered discrimination from their families, neighbors, and the community due to the disgusting odor of urine and the unremitting wetness they had been experiencing from an obstetric fistula:

*“You’ll face a lot of discrimination from some of your dads and moms, friends, and the community due to the disgusting odor and discolored clothes you wear. Even the kebele workers will not tell us as there is a meeting and we can’t participate.” **(Woman with fistula, 50 years).***

A similar woman with a fistula described her experiences of being discriminated against by family and neighbors:

*“My family refused to accept me as a functional person after this disease. They removed me from any community and family participation. I lived on my own in one house with a hunger for a long time. I couldn’t do marketing due to worry about people’s facial expressions. The Neighbors cover their noses and mouth and begin to speak about me once they observe me.” **(Divorced woman with fistula, 35 years).***

#### Enacted stigma

Similarly, many women with fistulas had reported being stigmatized by society:

*“What is shocking for me is when I go to church, people prefer talking about me rather than hearing the paster. I have a bad memory of the market; one of the women returned what her child bought from me after hearing that the material was mine. I lost a lot from the fistula! My beauty! My hope! and my lough! I am socially paralyzed.” **(Woman with fistula, 50 years).***

Another woman with fistula supplemented with her encounters of enacted stigma from society:

*“Our society (community) is the first to discriminate against us. When I pass nearby them on the road, they insult me with slang words saying ‘a urinating cow’ as they are saying to cows. Even though my cloth is dry, they know I am a victim, they cover their noses, expel their saliva, turn their face, do not want to move with me to the market area, and do not inform me when they go for social life (e.g., taking Injera to condolence).” (**Married woman with fistula, 30 years).***

#### Perceived stigma

Some of the women living with obstetric fistula perceived that they have been stigmatized by the community due to their fistula condition:

*“The odor of leakage, our low self-esteem, and the suspicion of being hated made us lose a lot. Oh! It is painful! painful! All eyes of people look at you, all their noses smell your urine, and all their ears and mouths hear and talk about you even though you are a single woman living in your home. I prefer loneliness not to seeing anyone. When people come to my home to reassure me, I prefer to insult and shout at them because I consider them as they came to see my smells and share my secret with others.” **(Woman with fistula, 50 years).***

Similarly, another woman with a fistula made a testimony of experiencing perceived stigma from a community:

*“You will be full of suspicion that even if people talk about anything and laugh, you will consider as they are talking about you even though they may not know about you. My thinking is affected and I will think as all people hate me; I isolate myself because of the low self-esteem I have and prefer to die (performing suicide).” (**Married woman with fistula, 30 years).***

#### Limitations on religious practices

Most of the women with fistula reported that they faced serious difficulties to practice their religious worshiping due to unrelieved dribblings of urine:

*“A lot of suffering was with this disease; which was continuous throughout the day and night. When you wake up every morning, you should have to hurry to clean up the area to remove the disgusting odor of urine. It has been difficult for me to go to church to pray with such a difficult condition.” **(Woman with fistula, 50 years).***

Concerning obstetric fistula’s disastrous obstacles to women’s spiritual worshiping and practice, one participant said:

*“…Women of Muslim religion followers were unable to go for worshipping in mosques with this problem due to their wettings from the dripping of urine. Therefore, they lived in isolation from religious areas and communities. I am not going to worship at the mosque due to this problem, but after cleaning my body and cloth I can attend mourning places.” **(Divorced woman with fistula, 25 years).***

#### Limitations on social life participation

Many women with fistula also reported that they encountered difficulties in engaging in social life:

*“Participating in social life is painful for us as we think of our odor always. For example, going to church, going to a marital ceremony, and coming to the salon to drink coffee with neighbors is difficult. The difficulty was socializing as before (e.g., drinking coffee, going to market, churches, meetings, etc.).” **(Woman with fistula, 50 years).***

Another participant also reported difficulty in participating and working in social life:

*“It becomes too tough to work as an ordinary person and to engage in social life since the leakage of urine will boom together with your motion. The big problem is being afraid of standing up from your sitting. Participating in social life is very difficult. For example, going to church, going to condolence, and going to marriage occasions are difficult.” **(Married woman with fistula, 30 years).***

Similarly, another woman with a fistula reported her difficult encounters to participate in social activities:

*In our community, people work and live together; they sell and buy in a group; they care for children and eat together. However, a woman with this problem cannot be seen as a woman; they consider as you are out of mind! After I face this fistula, I am out of all these activities.” **(Divorced woman with fistula, 35 years).***

### Consequences of fistula

Obstetric fistula has different consequences on the health of women with fistula. Almost all participants mentioned that women with fistula faced: internal suffering/ psychological problems, physical health problems, marital problems, and sexual and reproductive health problems. They noted that: they felt despair, failed to live the desired life, lost desire, satisfaction, and confidence in their marital and sexual health, were ashamed of their condition, with diminished self-esteem, had a painful life, felt bad, and preferred to die.

#### Psychological health problems

Many women living with obstetric fistulas encountered psychological health problems such as stress, depression, feeling bad, despair, worrying about their perceived and enacted stigma, and related internal suffering:

*“I have continuous mental stress about my health and the future life of my children. Previously, I had no problems. However, now I have incontinence of urine and feces. I am very depressed. Sometimes when I am alone, I think about my children’s care and my husband’s attitudinal changes due to my fistula. Thus, I am always worried about who takes care of my children if I die.” **(Separated multigravida woman with fistula, 25 years).***

Similarly, another woman with a fistula explained the effect of the fistula on her psychological health as:

*“Everybody covers their nose and mouth and starts to talk about me when they look at me. At that time, my mind is disturbed very much, I feel bad, lonely most of the time, and preferred to die. I couldn’t buy and sell due to fear of people’s facial expressions. I didn’t see any women like me before, who have the same problem. I always talk to myself about why I am the only woman who suffers from this problem. Why?…" **(Divorced woman with fistula,35 years).***

Obstetric fistula has exposed women with fistulas to worry about their enacted and perceived stigmas, which in turn aggravated their psychological health problems; one woman with a fistula explained this as:

*“Sometimes my friends come to my house to ask me but they stay far from me. When I go to a wedding or condolence, people cover their noses and mouth; talk about me, and try to stay far from me. I always worry about such encounters. These make me feel very bad and think about why I am the only person troubling with this problem. This provokes my feelings of sadness and depression; and leads me to cry most of the time. I also always think that all people talk only about me. Thus, I am afraid to see people and stay at home.” **(Married woman with fistula, 18 years).***

#### Physical health problems

Women living with obstetric fistula reported facing physical health problems including repair site infection, burning sensations, and foot drops:

*“…I fail to move and even to stand alone right away after delivery. The episiotomy area nurse cut was very pain full, nothing was done to treat the wound. I was very sick, I developed fever, headache, loss of appetite (I couldn’t eat and drink), and loss of sleep during the night. When I sit or try to use the toilet, I feel heaviness just like something is coming out.” **(Married woman with fistula, 18 years).***

A family member of the woman with a fistula reported the physical health consequences of the fistula as:

*“I have understood the severity of the fistula of my wife with continued stool & urinary incontinence. Additionally, there was an infection on the episiotomy site, full of bleeding and pus. Then, I tried my best to save her mind and life." **(Husband of a woman with fistula, 37 years).***

A woman living with obstetric fistula reported that she had been experiencing a burning sensation from her fistula condition:

*“…When I wash clothes, I experience a burning sensation in my vagina. It burns me like a flame in my vagina and inner thighs…” **(Woman with fistula, 30 years).***

Another woman with a fistula reported that she developed foot drops due to her fistula condition:

*“My legs are locked due to labor; I cannot go out, and even I have difficulty in standing. Starting from the commencement of this problem, I have experienced leg pain and difficulty walking.” **(Divorced woman with fistula,35 years).***

#### Sexual and reproductive health problems

Many women living with obstetric fistulas reported that they faced sexual and reproductive health problems:- lost feelings for sex and satisfaction, the difficulty of having a marriage and a baby:

*“Fistula makes you full of fear whilst you consider sexual life. It makes you different from other women. (e.g., vaginal secretion of urine decreases your feeling for sex). The big problem was thinking about sexual intercourse, having a marriage, and having a baby. This is to say, you will lose your confidence in talking with a male(boy), and the impact will make you lose your desire and satisfaction. You will also consider yourself as infertile and inferior to all women." **(Married woman with fistula, 30 years).***

Similarly, another woman with a fistula explained the effects of the fistula on her sexual health as:

*“…moreover, my fistula impacts my sexual lifestyle, I whinge that I’ve no longer had sexual intercourse due to the fact I confronted a fistula.” (**Multigravida woman with fistula, 40 years).***

#### Limitations to daily activities

Most of the women with obstetric fistula reported that they had faced difficulties to run day- to- day routine productive activities:

*“As a result of this problem, I can`t do routine activities like going to the market to buy or sell goods and going to the farming area. I only try to wash clothes and bake injera and sell, just to obtain money for my children`s daily food. My husband died While I used to be pregnant. I could not do more productive activities that would enable me to gain a better income. I have three children. I have paid sacrifices to grow my children without their father. You can see how much it is difficult (carrying)……. It is an additional burden.” **(Woman with fistula, 30 years).***

Another woman with a fistula described the effect of the fistula on her daily activities as:

*“If the woman faces a fistula, she cannot go to the market, mosque/church, cannot go anywhere outside the home. She fears sitting with other people. I didn’t leave home when I had been at home before coming here. My parents also did not allow me to go out of home because they fear people will insult me. I cannot lead my social lifestyle with the aid of going here and there. I have a health problem like a bad smell. I can’t work my daily activities, no participation in social life, and no sexual life at all.” **(Married woman with fistula, 20 years).***

#### Impaired marital status

Women with fistulas gave testimony that their marital status was impaired and most of them divorced after their marriage due to fistula:

*“…it is polygamy based on my decision. I have badly suffered from this condition for five years. I stayed one and a half years with my family, leaving my husband alone… then he asked me to back to my home. I back home thinking about his loneliness. … after four years he told me as he needs to have a child; I allowed him to marry, and I left his home. Of course, due to this problem, my husband married another wife over me; due to the marriage, I had these challenges; I am insulted a lot." (**Multigravida woman with fistula, 30 years).***

A health care provider also added to the effects of fistula on women’s marital status as:

*“Majority of the women will divorce after fistula because of the problem and many of them were primigravida mothers. The majority of the babies born from mothers with fistula will die soon. Therefore, since there are no ties to continue their marriage, so they divorce easily.” **(Health officer, Male, 42 years).***

### Coping mechanisms with obstetric fistula problems

**Fig 1:**
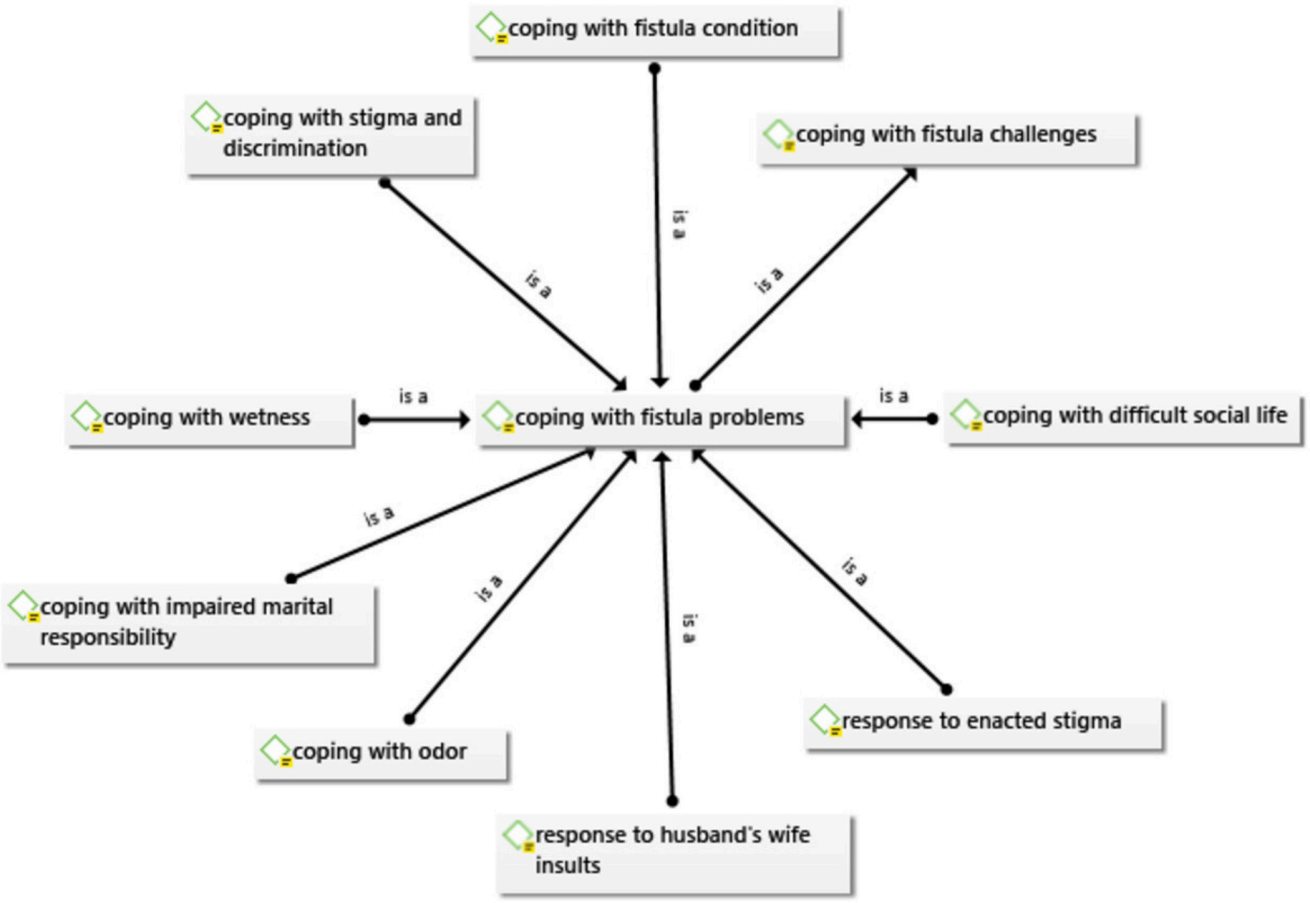
Coping mechanisms with obstetric fistula problems.

Women with obstetric fistula stated the use of various coping mechanisms for their encountered fistula troubles such as: coping with fistula condition/disease, coping with difficult social life, odors, wetness, and marital responsibilities (Fig 1).

#### Coping with the fistula condition/disease as all

Participants reported that they coped with their fistula condition: through the support gotten from their families, by indulging in busy homework activities to forget the disease, going to different spiritual and praying areas, taking traditional medicines, drinking alcohol, and discussing their disease condition with other similar women:

*“The support from my husband, children, and parents gave me the strength to cope with the disease. To make my mind busy, I was occupied with a lot of homework throughout the day. Going to different traditional praying areas gave me little mental rest even though not permanent relief. E.g., when I go to someone’s home and take sunstroke medicine, I sleep peacefully. Once in a while, once I sense despair, I drink alcohol the whole night to forget about all matters. After knowing about this disease and starting meeting previous victims of fistulas in my kebeles, getting them, discussing with them, and staying with them all helped me a lot to forget the disease. This also helped me to be ready to go for treatment and gave me hope to live in life.” **(Woman with fistula, 50 years).***

#### Coping with a difficult social life

Regarding their coping mechanisms to the difficult social life, participants mentioned using strategies like: living alone in the forest, isolating themselves from community participation, crying and praying to God, not talking about their condition with anyone, hiding their fistula condition by keeping their hygiene every day, and by proscribing the quantity of water they drink::

*“Your family may display you awful facial features and bad speech. Even, I had decided to live in the forest like wild animals not to look and hear anyone. I had also isolated myself from participating in community meetings and celebrations (e.g., kebele meetings, weddings, and mourning). Tolerance… hoping that I will get cured of it one day if it is the will of God. Sometimes I cry; sometimes I pray and leave them believing that God will cure me of this problem one day. Other times, I prefer not hearing what has been said, instead, I am praying and visiting health facilities." **(Married woman with fistula, 30 years).***

Another woman with fistula reported on coping mechanisms to difficult social life through hiding self, restricting drinking water, and keeping personal hygiene:

*“I didn`t disclose my condition to my neighbors. They didn`t know the situation I was in, because I keep my hygiene every day by washing my clothes and taking a shower. Sometimes to hide my fistula problem, I restrict myself from drinking water. Therefore, I get some relief from dribbling urine, bad odor, and fear of individuals’ perceptions. I never told to anybody since the fistula is shameful. I hide it not to be stigmatized by my neighbors and others.” **(Woman with fistula, 30 years).***

#### Coping with wetness and odor

Participants reported that they coped with wetness and odor by repeatedly washing their bodies and clothes, changing clothes frequently, and by wearing two up to three clothes at a time:

*“I cope with wetness and odor: by repeatedly washing, wearing trousers, changing clothes three to four times per day, going to the river to wash my clothes more than three times per week.” **(Multigravida woman with fistula, 30 years).***

Another woman with a fistula reported that she had been cleaning the area and wearing many folds of clothes at a time to cope with wetness and odor:

*“When I wake up every morning, I should have to hurry to clean up the area to remove the disgusting odor of urine. When I go out, I should have to wear two-three clothes to hide the wetness & odor which is out of my control but make me ashamed. Worrying about the odor of the urine makes me drier wood.” **(Woman with fistula, 50 years).***

Furthermore, concerning how women with fistulas cope with wetness and odor, the health care provider stated:

*“Patients with fistula take few or not take water/fluid to minimize the amount of urine. It minimizes the amount of urine. However, if the urine passes out it’s very concentrated and has an offensive smell. Thus, mothers should take adequate water or fluid to minimize the smell. Again, it helps also her treatment and to improve her health. You can see our ward, you can’t imagine how it is free from smelling this much. This is because we advised them to take adequate fluids and water, but if you see our patients when they first come to the hospital they have a very offensive smell due to poor hygiene and accumulated and concentrated urine they have.” **(BSc Nurse, Female, 42 years).***

#### Coping with impaired marital responsibility

Some participants mentioned coping with the inability to commit marital responsibilities through allowing their husband to marry a new wife; tolerating all insults and challenges, and trying to participate in productive activities:

*“I allowed my husband to marry another wife considering my problems and difficulties … before that, he is good and cared for me, now he … has no concern for me. I feel very bad and to leave the house/divorce. I passed those years…caring for myself… for the last three years. The problem becomes worsen to me and known to his new wife and others… I am still with her tolerating all insults and challenges.” **(Multigravida woman with fistula, 30 years).***

Another woman with a fistula reported that she coped with impaired marital responsibility through participating in productive activities:

*“When my husband left me, I tried to farm and deal with onions, corn, and other things present in our area. However, it has been very difficult for me to work while living with this problem. Most of the time, I stay at home hungry as you look at my physical; I lost weight; and my physical appearance has changed very much. You see me now after I have got much improvement since I came here.” **(Divorced woman with fistula, 35 years).***

## Discussion

This study was an in-depth interview-based phenomenological study that explored the challenges and coping mechanisms amongst women living with obstetric fistulas in five fistula treatment facilities in Ethiopia. Through thematic analysis, three themes such as painful social life, consequences of fistula, and coping mechanisms with fistula problems were developed to answer the research question. Participants gave their testimony that women with fistula faced many challenges. These include painful social life, marital problems, sexual and reproductive health problems, stigma, internal suffering or psychological health problems, and physical health problems. They reported different coping mechanisms including coping with a difficult social life, coping with fistula conditions, coping with impaired marital responsibility, and coping with wetness and odors.

In this study, women with fistula faced diverse demanding situations together with a painful/hard social lifestyle at the same time as dwelling with fistula. Similar studies in Malawi, Tanzania, Niger, and Nigeria showed that their families and community members rejected women with fistulas. Such women have been: pushed away from attending spiritual observances or community gatherings; lost social support, and highly stigmatized, marginalized, and ostracized by their husbands, families, and community because of their obstetric fistula condition [17, 35–38]. Studies show that throughout most African countries, most of the women living with obstetric fistulas experienced almost similar difficult social life and consequences of fistula [39–44]. However, studies in Malawi, Ghana, and Tanzania show that many women with fistula experienced stigma, isolation, disgrace, decreased feelings of worth, and psychological trauma due to their incapability to fulfill the culturally ascribed marital roles and social expectancies of them as ladies, wives, and mothers (the cultural importance of childbearing and motherhood) [17, 38, 45].

Similarly, in this study, fistula women reported that fistula had devastating health problems in their sexual and reproductive health, psychological health, physical health, routine household activities, and marital responsibilities. This confirms findings from sub-Saharan Africa on the consequences of obstetric fistula on women who endured the condition including physical challenges of losing body control, women’s social and family relationships, and the challenges of losing financial gaining. Obstetric fistula has also a far-reaching consequence on women’s physical well-being, social and marital relationships, mental health, and economic capacity [24]. This collaborates also with a global public health report on obstetric fistulas that women with fistulas who usually have incontinence are equally affected and live a stigmatized life with social, economic, psychological, reproductive, and sexual repercussions [7]. A similar study in Nigeria shows that many women and girls with obstetric fistulas face many psychological problems such as humiliation, abandonment, stigmatization, loneliness, separation from their husbands, and hopelessness [16]. Other studies in developing countries show that victims of obstetric fistula often feel that their future is frightening. They uncertain about having partners; marriage, sex, becoming pregnant, giving birth, and reintegrating back into their local communities [17–19]. Related to their physical health problems, the previous study shows that inconsistencies of urine due to obstetric fistula affect the self-esteem of women with fistulas and might bring about great secondary morbidity, incapacity, and infertility [21].

In this study, we found that women with fistula have used different coping mechanisms: going to spiritual sites, drinking alcohol, through the support gotten from their families, and discussing their disease condition with other similar women to cope with their fistula disease. Separating self from community participation and living alone in the forest to cope with a painful social life. Preserving their hygiene every day and minimizing the amount of ingesting water, wearing many garments at a time to cope with wetness and odors. Allowing their husband to marry a new wife to cope with impaired marital responsibilities. This collaborates with studies conducted in northwest Ethiopia and Somalia [46, 47]. Moreover, these findings support other studies conducted in Indonesia that participants used strategies, including “meaning-making”- a psychological process of accepting their disease status that helped their attitudes to think positive about life, self-reliance, religious/spiritual, openly talking and sharing their psychological state of mind with families, and awareness of the importance of family supports as a mechanism to cope with the challenging conditions of their disease; cognitive or acceptance strategies, knowledge of health condition, family relationship and support to cope with psychological challenges; social withdrawal to cope with social impacts, stigma, and discrimination [48, 49]. A similar study in Mali showed that women with fistulas linked their urinary inconsistence to sexually transmitted infections to cope with a painful social life; particularly to avoid stigma [40]. In West Africa and Sudan to cope with the disease, participants used the phrase, "It is God’s will." "This indicated that the obstetric fistula turned into their fate or changed into destined by way of God". Hence, no matter how bad it was, they had to accept it [40, 43]. Another study in Malawi shows that women with fistulas who remained with their first husbands: retained social, emotional, and financial support from their husbands. This is because, according to the Yao community, a factor that may be conducive to men supporting wives with fistulas is the tradition of polygamy. This means that the husband may find sexual fulfillment through a second wife while supporting a wife with an obstetric fistula. In contrast, regarding their coping with impaired marital status, the study shows that some fistula survivors remarried men who were fully aware of their condition [38]. In East Africa, to cope with impaired marital responsibility, participants thought that women with fistulas could not bear children, and therefore many lost the hope of bearing children in the future [17]. Other studies in East–Africa and Ghana show that women with fistulas wrapped themselves up in old dresses; they used frequent washing of bodies and clothes and perfumes to cope with the wetness of urine and odors [17, 42].

### Necessity and practice implications of the study

Overall, the results of the current study suggest that exploring the challenges obstetric fistula women have been facing and the coping mechanisms used is a cue for action for decision-makers to prioritize the availability, accessibility, and early utilization of obstetric fistula surgery and post-operative care in all settings for all women living with obstetric fistula for restoring their holistic health and dignity. The findings of the study may also be used as a source of information for designing programs and strategies for community awareness creation about fistulas for the prevention of enacting stigma and discrimination against such women; for educating women living with obstetric fistulas on appropriate coping mechanisms; and on the detrimental consequences of some of the coping mechanisms being used by them such as living alone in a forest, restricting the amount of drinking water, taking traditional medicines, drinking alcohol, and allowing a husband to marry a new wife. It may be important information for health care practitioners dealing with women living with obstetric fistulas for understanding the extent to which such women’s overall health has been devastated by obstetric fistulas and to act accordingly for alleviating those challenges women have been facing. It may also be used as baseline data for any researcher wanting to conduct a grounded theory on a similar topic.

### Strengths and limitations

This study employed triangulation methods, through the research team’s independent coding of the data, and the data were collected from women with fistula and key informants, which contributed to a more understanding of the phenomenon (challenges and coping mechanisms). Using purposive sampling enabled us to get individuals that provided rich, relevant, and numerous facts pertinent to the research question. The outstanding strength of this study is that it ensured the trustworthiness of the collected data through the application of strategies such as credibility, transferability, dependability, and conformability. Moreover, the participants were interviewed in a separate place without any interference supplemented with further probes, field notes, and audio recordings, which helped us to explore fully the data in depth. The limitation of this study is the inability to employ a maximum variation sampling technique with key informants due to feasibility problems. This means, that due to the unavailability and inaccessibility of some of the key informants during the data collection period, the study has not included enough key informants from different facilities with different background features. Another limitation of the study is that group debriefing and repeat interviews were not conducted with study participants.

## Conclusion

This study explored that women with obstetric fistulas encountered challenges such as a painful social life, impaired marital status, and psychological, physical, sexual, and reproductive health problems. Women living with fistula have used coping mechanisms such as—going to spiritual sites and drinking alcohol to cope with their fistula disease; separating themselves from community participation and living alone in the forest to cope with a painful social life; keeping their hygiene every day, restricting the amount of drinking water, and wearing many clothes at a time to cope with wetness and odors, and allowing their husband to marry a new wife to cope with the impaired marital responsibilities. The findings highlight an urgent need for strategies to battle social stigma and discrimination experienced by women with fistulas through communities’ awareness creation about obstetric fistulas to enhance the social life, dignity, and overall well-being of such women. The result also indicates a need to early link women with fistulas to treatment centers for their earlier access to repairs to halt all potential consequences of obstetric fistula on their health. Importantly, the study demonstrates a need to support such women with their coping mechanisms with obstetric fistula problems and psychological support is paramount. In turn, policy-makers at all levels should also prioritize the availability, quality, accessibility, and acceptability of obstetric fistula surgery in all settings for women with obstetric fistula for restoring their sexual, reproductive, physical, psychological, and social health.

## Supporting information

S1 Table COREQ-checklist. (DOCX)

S2 Table In depth interview guide. (DOCX)

S1 Text Women with fistulas’ data. (PDF)
