# Supplementary material for: Challenges and coping mechanisms among women living with unrepaired obstetric fistula in Ethiopia: A phenomenological study
Source: PLoS One. 2022 Sep 29;17(9):e0275318. doi: 10.1371/journal.pone.0275318 (PMC9522016; doi:10.1371/journal.pone.0275318)
Supplement: S1 Table — (DOCX) [file pone.0275318.s001.docx]

**Manuscript: Challenges and coping mechanisms among women living with unrepaired obstetric fistula in Ethiopia: a phenomenological study**

**Consolidated criteria for reporting qualitative studies (COREQ): 32-item checklist**

Developed from:

Tong A, Sainsbury P, Craig J. Consolidated criteria for reporting qualitative research (COREQ): a 32-item checklist for interviews and focus groups. *International Journal for Quality in Health Care*. 2007. Volume 19, Number 6: pp. 349 – 357

| **No. Item** | | **Guide questions/description** | | **Reported on Page #** |
| --- | --- | --- | --- | --- |
| **Domain 1: Research team and reﬂexivity** | |  | |  |
| *Personal Characteristics* | |  | |  |
| 1. Inter viewer/facilitator | | Which author/s conducted the interview or focus group? | | Page 7 / paragraph 2  (Line 138-139) |
| 2. Credentials | | What were the researcher’s credentials? E.g. PhD, MD | | Page 7 / paragraph 2  (Line 140) |
| 3. Occupation | | What was their occupation at the time of the study? | | Page 7 / paragraph 2  (Line 140) |
| 4. Gender | | Was the researcher male or female? | | Page 7 / paragraph 2  (Line 139) |
| 5. Experience and training | | What experience or training did the researcher have? | | Page 7 / paragraph 2  (Line 141-142) |
| *Relationship with participants* | |  | |  |
| 6. Relationship established | | Was a relationship established prior to study commencement? | | Not reported on page  **Yes** |
| 7. Participant knowledge of the interviewer | | What did the participants know about the researcher? e.g. personal goals, reasons for doing the research | | Page 29 / paragraph 1  (Line 602-603)  Participants were briefed on the purpose of the study and understood it. Ethical had granted, participants’ information sheet was read for them prior to giving their written informed consent to be involved in the study |
| 8. Interviewer characteristics | | What characteristics were reported about the inter viewer/facilitator? e.g. Bias, assumptions, reasons and interests in the research topic | | Not reported on page  Yes |
| **Domain 2: study design** |  | |  | |
| *Theoretical framework* |  | |  | |
| 9. Methodological orientation and Theory | What methodological orientation was stated to underpin the study? e.g. grounded theory, discourse analysis, ethnography, phenomenology, content analysis | | Page 5 / paragraph 2  (Line 109-110) | |
| *Participant selection* |  | |  | |
| 10. Sampling | How were participants selected? e.g. purposive, convenience, consecutive, snowball | | Page 6 / paragraph 2 (Line 118- 121, 127-128) | |
| 11. Method of approach | How were participants approached? e.g. face-to-face, telephone, mail, email | | Page 7 / paragraph 2  (Line 138) | |
| 12. Sample size | How many participants were in the study? | | Page 6 / paragraph 4  (Line 118-119) | |
| 13. Non-participation | How many people refused to participate or dropped out? Reasons? | | **None** | |
| *Setting* |  | |  | |
| 14. Setting of data collection | Where was the data collected? e.g. home, clinic, workplace | | Fistula treatment center. Page 5 / paragraph 1  (Line 98-99) | |
| 15. Presence of non-participants | Was anyone else present besides the participants and researchers? | | **No** | |
| 16. Description of sample | What are the important characteristics of the sample? e.g. demographic data, date | | Page 6, paragraph 2, Line 121,Table 1 & 2 (page 9 & 10) | |
| *Data collection* |  | |  | |
| 17. Interview guide | Were questions, prompts, guides provided by the authors? Was it pilot tested? | | Page 6-7 / paragraph 3 (Line 130-136) | |
| 18. Repeat interviews | Were repeat inter views carried out? If yes, how many? | | **No** | |
| 19. Audio/visual recording | Did the research use audio or visual recording to collect the data? | | Page 7 / paragraph 2 (Line 145) | |
| 20. Field notes | Were ﬁeld notes made during and/or after the interview or focus group? | | Page 7 / paragraph 2 (Line 146) | |
| 21. Duration | What was the duration of the inter views or focus group? | | Page 7 / paragraph 2 (Line 144-145) | |
| 22. Data saturation | Was data saturation discussed? | | Page 6 / paragraph 2, line 122-127, Page 7/paragraph 2 line 147-148 | |
| 23. Transcripts returned | Were transcripts returned to participants for comment and/or correction? | | **No** | |
| **Domain 3: analysis and ﬁndings** |  | |  | |
| *Data analysis* |  | |  | |
| 24. Number of data coders | How many data coders coded the data? | | Six including the author, page 8 paragraph 2, line 165-169 | |
| 25. Description of the coding tree | Did authors provide a description of the coding tree? | | Page 8 / paragraph 2 (Line 167-170) | |
| 26. Derivation of themes | Were themes identiﬁed in advance or derived from the data? | | Page 7 / paragraph 2 (Line 169-170, 157-158)Themes were derived from the data | |
| 27. Software | What software, if applicable, was used to manage the data? | | Page 8 / paragraph 2 (Line 170-171), **Atlas. ti** | |
| 28. Participant checking | Did participants provide feedback on the ﬁndings? | | **No** | |
| *Reporting* |  | |  | |
| 29. Quotations presented | Were participant quotations presented to illustrate the themes/ﬁndings? Was each quotation identiﬁed? e.g. participant number | | Themes and sub-themes were presented and described supported by participant quotes. | |
| 30. Data and ﬁndings consistent | Was there consistency between the data presented and the ﬁndings? | | **Yes,** see Table 3 per findings presented from page 11-23. | |
| 31. Clarity of major themes | Were major themes clearly presented in the ﬁndings? | | Yes, Page 10 Table 3, Line 193-195, Page 11-line 196, page 15-line 279, page 20-line 383 | |
| 32. Clarity of minor themes | Is there a description of diverse cases or discussion of minor themes? | | **Yes** ,Page 11-23 /  Page 11-line 200  Page 12-line 215 & 229  Page 13-line 246  Page 14-line 260  Page 15-line 286  Page 16-line 313  Page 17-line 335  Page 18-line 350  Page 19-line 368  Page 20-line 388  Page 21-line 403  Page 22-line 425  Page 23-line 447 | |
