# Supplementary material for: Challenges and coping mechanisms among women living with unrepaired obstetric fistula in Ethiopia: A phenomenological study
Source: PLoS One. 2022 Sep 29;17(9):e0275318. doi: 10.1371/journal.pone.0275318 (PMC9522016; doi:10.1371/journal.pone.0275318)
Supplement: S2 Table — (DOCX) [file pone.0275318.s002.docx]

**In depth interview guide for women with obstetric fistula**

| **PART I:- Background information** |
| --- |
| - Transcript code: - District: - Kebele/residence: - Date : - Start time - Finish time: - Interviewer:   : |

| No | Questions | Response options | Skip |
| --- | --- | --- | --- |
|  | What is your present age? | 1. Year___________ |  |
|  | What is your residence? | - - - 1. Urban       2. Rural |  |
|  | What is your marital status? | 1. Never married 2. Married 3. Cohabiting/living together 4. Divorced/Separated 5. Widowed |  |
|  | What is your occupation | 1. Non-Government employee 2. Government employee 3. Farmer 4. Merchant 5. Student 6. Housewife 7. Others specify---------------- |  |
|  | What is your educational level? | - - - 1. Can’t read and write       2. Read and write       3. Primary schools       4. Secondary and preparatory schools       5. College/university |  |

**PART II**

**Lived experiences**

1. How women with obstetric fistula in your area face different problems related to their fistula?
2. What are your experiences with such problems after your obstetric fistula so far?

**…. Probe,** from your husband, your family, your society and community

**…. Probe,** what you lost from (husband, families, society, and community) due to your fistula? Why/how you lose?

1. Have you ever faced discrimination/stigmatization?

**….Probe,** how can you explain discrimination/stigmatization from your (husband, family, society, community)?

1. How can you explain the impacts of obstetric fistula on your life?

**…Probe**, related to your marriage?

**….Probe**, related to your sexual life, reproductive life and marital life?

1. How you cope with those encountered problems due to your fistula?

**…Probe,** with your illness**,** wetness and odors?

**In depth interview guide for key informants**

How women with obstetric fistula in your area face different problems related to their fistula?

What do they face while living with obstetric fistula?

**…. Probe**, from their husband, their family, from society and community

**…. Probe**, what do they lose from (husband, families, society, and community) due to obstetric fistula? Why?

Do they face discrimination/stigmatization?

**….Probe**, from their (husband, family, society, community)?

How do you explain the impact of obstetric fistula on their life?

**…Probe**, related to their marriage?

**….Probe**, related to their sexual life, reproductive life and marital life?

How do women with obstetric fistula cope with those encountered problems

**…Probe**, with their illness, wetness and odors?

***Thank you!***

Data collector

Name ________________ Signature _______________

Supervisor

Name ________________ Signature ____________

**NB:** Part I (background information) of the interview guide is similar both for women with fistula and key informants.
