# Supplementary material for: Challenges and coping mechanisms among women living with unrepaired obstetric fistula in Ethiopia: A phenomenological study
Source: PLoS One. 2022 Sep 29;17(9):e0275318. doi: 10.1371/journal.pone.0275318 (PMC9522016; doi:10.1371/journal.pone.0275318)
Supplement: S1 Text — (PDF) [file pone.0275318.s003.pdf]

Project: Women with fistulas' data

Report created by user on 8/19/2022

Code Report

All (76) codes

○ challenges after fistula

Comment: by toshiba

> *surgery not done*

2 Quotations:

23:9 Concerning myself, I faced different challenges due to my fistula case (494:580) - D 23: PD-Client 6

Concerning myself, I faced different challenges due to my fistula case from my husband.

1 Codes:

○ challenges after fistula

24:25 This leaking of urine I experienced is not like a leaking of urine I h (2912:3108) - D 24: PD-Client 7

This leaking of urine I experienced is not like a leaking of urine I have seen on other women; the amount is scanty. The big challenge is that I can't control my urine and feces just when it comes.

1 Codes:

○ challenges after fistula

○ challenges after operation

Comment: by toshiba

> *no body visits me from family*

0 Quotations

○ challenges from husband

Comment: by toshiba

> *insult, hide , divorce , need to marry other, lack of happiness and love for me*

3 Quotations:

23:10 He ignored me and married another wife in addition to me. (582:639) - D 23: PD-Client 6

He ignored me and married another wife in addition to me.

1 Codes:

○ challenges from husband

23:11 Sometimes, I have experienced stigma and discrimination from my husban (641:712) - D 23: PD-Client 6

Sometimes, I have experienced stigma and discrimination from my husband.

1 Codes:

○ challenges from husband

24:16 Since this urine linkage is happened, my husband never came to me and (1652:1791) - D 24: PD-Client 7

Since this urine linkage is happened, my husband never came to me and he divorced me. Now, he has married another woman and living with her.

2 Codes:

○ challenges from husband / ○ consequences of fistula on marital

---

○ consequences of fistula

Comment: by toshiba

> *outcomes of living with fistula, wetness and odor of out of control, being ashamed. unable to control urine*

33 Quotations:

1:10 A lot of suffering were with these disease; which was continuous throu (657:783) - D 1: PD-Client 1

A lot of suffering were with these disease; which was continuous throughout the day and night, when you wake up every morning

1 Codes:

○ consequences of fistula

1:12 the disgusting odor of the urine (843:874) - D 1: PD-Client 1

the disgusting odor of the urine

1 Codes:

○ consequences of fistula

1:15 the wetness & odor which is out of you control but make you ashamed (965:1031) - D 1: PD-Client 1

the wetness & odor which is out of you control but make you ashamed

1 Codes:

○ consequences of fistula

1:16 the wetness & odor which is out of you control but make you ashamed ” (964:1090) - D 1: PD-Client 1

the wetness & odor which is out of you control but make you ashamed ” yaaddon foolii fincaaniicha mukaa gogaatti sii hanbisa”

1 Codes:

○ consequences of fistula

1:17 Participating in-social life is painful for us (1097:1144) - D 1: PD-Client 1

Participating in-social life is painful for us

3 Codes:

○ consequences of fistula / ○ difficult social life / ○ painful social life

1:18 since we think of our odor always. (1146:1179) - D 1: PD-Client 1

since we think of our odor always.

2 Codes:

○ consequences of fistula / ○ stressed with odours

1:25 your fear will put you in stress (1593:1624) - D 1: PD-Client 1

your fear will put you in stress

1 Codes:

○ consequences of fistula

1:40 I lost a lot from fistula! my beuty!,my hope!, my lough !socialy paral (2794:2870) - D 1: PD-Client 1

I lost a lot from fistula! my beuty!,my hope!, my lough !socialy paralyzed,

1 Codes:

○ consequences of fistula

1:42 the odor of the leakage, our low self steem, over suspicion of being h (2966:3063) - D 1: PD-Client 1

the odor of the leakage, our low self steem, over suspicion of being hated made us to lose a lot.

1 Codes:

○ consequences of fistula

1:45 you will face a lot of stigma and discrimination from some of your par (3184:3350) - D 1: PD-Client 1

you will face a lot of stigma and discrimination from some of your parents, neighbors and the community due to the disgusting odor and discolourated clothes you wear.

1 Codes:

○ consequences of fistula

1:59 Fistula made you to be full of fear when you think of sexual life and (5453:5635) - D 1: PD-Client 1

Fistula made you to be full of fear when you think of sexual life and it makes you different from other women (e.g. vaginal secretion with urine) which decrease your feeling for sex.

2 Codes:

○ consequences of fistula / ○ effects of fistula on sexual reproductive health

2:7 which when I thought about it get sick. (449:487) - D 2: PD-Client 2

which when I thought about it get sick.

1 Codes:

○ consequences of fistula

2:8 Fistula made us to fail physicaly, socialy and psychologicaly ingenera (489:560) - D 2: PD-Client 2

Fistula made us to fail physicaly, socialy and psychologicaly ingeneral.

1 Codes:

○ consequences of fistula

2:10 afraid the leakage,the smell and the face of people you will see (630:694) - D 2: PD-Client 2

afraid the leakage,the smell and the face of people you will see

1 Codes:

- ○ consequences of fistula

2:11 Not only these, It continues with the burnning sensation on urination (697:819) - D 2: PD-Client 2

Not only these, It continues with the burnning sensation on urination and the eyes of people who looks at your wet clothes.

1 Codes:

- ○ consequences of fistula

2:12 It was to difficult to work as normal person since the leakage of urin (838:1039) - D 2: PD-Client 2

It was to difficult to work as normal person since the leakage of urine will increase with your movement and the big problem is afraid of standing from your sitting"Libsishin atamgniwum, tisakekyalesh"

1 Codes:

- ○ consequences of fistula

2:15 Participating in-social life is painful for us since we think of our o (1216:1520) - D 2: PD-Client 2

Participating in-social life is painful for us since we think of our odor always.for example(going to church, "lekso and serg",even coming to salon to drink coffee with your neigh-bours is difficult and you will prefer lonliness and even to have aseparate home in the forest inorder not to see any one.

1 Codes:

- ○ consequences of fistula

2:19 Your thinking also affected (1702:1728) - D 2: PD-Client 2

Your thinking also affected

1 Codes:

- ○ consequences of fistula

2:22 low selfsteem (1818:1831) - D 2: PD-Client 2

low selfsteem

1 Codes:

○ consequences of fistula

2:29 It still continues: our society (community) is the first to dicriminat (2777:2851) - D 2: PD-Client 2

It still continues: our society (community) is the first to dicriminate us

1 Codes:

○ consequences of fistula

2:31 I lost a lot from fistula! My first marriage!, my beuty!,my hope!,my l (3211:3304) - D 2: PD-Client 2

I lost a lot from fistula! My first marriage!, my beuty!,my hope!,my lough !socialy paralyzed,

1 Codes:

○ consequences of fistula

2:33 the odor of the leakage,our low selfsteem, (3400:3441) - D 2: PD-Client 2

the odor of the leakage,our low selfsteem,

1 Codes:

○ consequences of fistula

2:35 The big problem which will make you hopeless and to hate your self is (3580:3683) - D 2: PD-Client 2

The big problem which will make you hopeless and to hate your self is is the stigma and discrimination!

1 Codes:

○ consequences of fistula

2:52 I haven’t seen him after all .Then, when I came back, he married anoth (5473:5581) - D 2: PD-Client 2

I haven’t seen him after all .Then, when I came back, he married another women eventhough we didn’t divorced.

2 Codes:

○ consequences of fistula / ○ problems due to fistula

2:53 The big problem was, when you thought about sexual intercourse,having (5590:5817) - D 2: PD-Client 2

The big problem was, when you thought about sexual intercourse,having marriage,having baby; this is to say,you will loose your confidence of talking with male(boy) , the impact will make you to loose your desire and satisfaction

3 Codes:

○ consequences of fistula / ○ effects of fistula on sexual reproductive health / ○ problems due to fistula

2:76 Since we are irritable and easy to cry to things, (8630:8679) - D 2: PD-Client 2

Since we are irritable and easy to cry to things,

3 Codes:

○ consequences of fistula / ○ problems due to fistula / ○ response to fistula problems

2:83 they are suffering from both physical and psychological problem (9345:9407) - D 2: PD-Client 2

they are suffering from both physical and psychological problem

1 Codes:

○ consequences of fistula

5:15 I have understood the severity of fistula on my Wife (stool& urine inc (1506:1730) - D 5: PD-Husband of the patient

I have understood the severity of fistula on my Wife (stool& urine incontinence) & then tried my best to save her mind and life. In addition to the incontinence, there Was infection on episotomy site full of bleeding and pus

2 Codes:

○ consequences of fistula / ○ problems due to fistula

18:15 After delivery urine starts to leak out (1879:1917) - D 18: PD-Client 4

After delivery urine starts to leak out

3 Codes:

○ consequences of fistula / ○ problems due to fistula / ○ problems experienced after first birth

21:31 but if the urine comes out its very concentrated and have offensive s (6250:6327) - D 21: PD-BSc Nurse

but if the urine comes out its very concentrated and have offensive smelling.

1 Codes:

○ consequences of fistula

21:36 if you see our patient when they first come to hospital they have very (7148:7303) - D 21: PD-BSc Nurse

if you see our patient when they first come to hospital they have very offensive smell due to poor hygiene and accumulated and concentrated urine they have.

1 Codes:

○ consequences of fistula

25:28 I suffer from burning sensation in my vagina due to continuous leakage (4768:4848) - D 25: PD-Client 8

I suffer from burning sensation in my vagina due to continuous leakage of urine.

1 Codes:

○ consequences of fistula

33:6 I faced with so many problems, (699:728) - D 33: PD-Client 11

I faced with so many problems,

2 Codes:

○ consequences of fistula / ○ problems due to fistula

○ consequences of fistula on daily cativity

Comment: by toshiba

> *can't do farming, going to market*

3 Quotations:

25:11 As a result of this problem, I can`t do routine activities like go to (1252:1594) - D 25: PD-Client 8

As a result of this problem, I can`t do routine activities like go to market to buy or sell goods, go to farming area. I only trying to wash clothes and baking injera and sell, just to obtain the money for my children`s daily food. When I wash clothes, I feel burning session in vagina. It burns me like a flame in my vagina and inner thighs.

1 Codes:

○ consequences of fistula on daily cativity

25:13 I have payed scarification to grow my children without their father. y (1790:1928) - D 25: PD-Client 8

I have payed scarification to grow my children without their father. you can see how much it is difficult(carrying)……. It is double burden.

1 Codes:

- ○ consequences of fistula on daily cativity

31:6 They faced with very difficult problems, for instance women with urine (382:595) - D 31: PD-Client 10

They faced with very difficult problems, for instance women with urine drippings cannot able to work and struggle to survive outside being exposed to surrounding peoples as they ashamed having contact with peoples.

2 Codes:

- ○ consequences of fistula on daily cativity / ○ consequences of fistula on social life

---

○ consequences of fistula on family

Comment: by toshiba

> *if client suffered the family are also in danger of stigmatization*

2 Quotations:

1:57 There was no problem on my family including my husband (5287:5340) - D 1: PD-Client 1

There was no problem on my family including my husband

1 Codes:

- ○ consequences of fistula on family

17:32 Because, if she suffered the whole family will be in danger. (5317:5376) - D 17: PD-Health officer

Because, if she suffered the whole family will be in danger.

1 Codes:

- ○ consequences of fistula on family

○ consequences of fistula on marital

Comment: by toshiba

> *not able to meet with husband*

13 Quotations:

17:17 Majority of the women will divorce after fistula because this problem (2945:3075) - D 17: PD-Health officer

Majority of the women will divorce after fistula because this problem and many of them were primigravida mothers were the victimed.

1 Codes:

○ consequences of fistula on marital

18:21 My husband left me! he told me as he didn't want me longer with this p (2512:2588) - D 18: PD-Client 4

My husband left me! he told me as he didn't want me longer with this problem!

1 Codes:

○ consequences of fistula on marital

18:28 Yes, I lost my marriage. Due to this disease x2 (3204:3250) - D 18: PD-Client 4

Yes, I lost my marriage. Due to this disease x2

1 Codes:

○ consequences of fistula on marital

24:16 Since this urine linkage is happened, my husband never came to me and (1652:1791) - D 24: PD-Client 7

Since this urine linkage is happened, my husband never came to me and he divorced me. Now, he has married another woman and living with her.

2 Codes:

○ challenges from husband / ○ consequences of fistula on marital

24:21 In addition, my husband had married another wife due to my obstetric f (2391:2466) - D 24: PD-Client 7

In addition, my husband had married another wife due to my obstetric fistula

1 Codes:

○ consequences of fistula on marital

24:27 As a result of my fistula, I lost my marriage… my husband leaves me al (3301:3416) - D 24: PD-Client 7

As a result of my fistula, I lost my marriage… my husband leaves me alone and went out from home with four children.

1 Codes:

○ consequences of fistula on marital

25:21 I have been influenced a lot as a result of my fistula. For instance, (3840:4127) - D 25: PD-Client 8

I have been influenced a lot as a result of my fistula. For instance, I want be married and also individuals have been asking me for marriage, but now, due to this problem I can't do that. If I marry, this person can support me in all dimensions (economy, in growing children and so on).

1 Codes:

- ○ consequences of fistula on marital

25:35 Different persons asked to marry me but I refuse them because of my fi (6429:6704) - D 25: PD-Client 8

Different persons asked to marry me but I refuse them because of my fistula. I said to them "I don`t want to be marry you because I am growing my children." the reason a lay them is I fear that the person I marry may be leave me after he see my fistula. That is why I refused

1 Codes:

- ○ consequences of fistula on marital

27:8 On my own, the problem I complain due to fistula is that I had missed (393:473) - D 27: PD-Client 9

On my own, the problem I complain due to fistula is that I had missed my marriage

1 Codes:

- ○ consequences of fistula on marital

27:15 Due to impact of stigma and discrimination as a result of fistula, I m (1321:1437) - D 27: PD-Client 9

Due to impact of stigma and discrimination as a result of fistula, I missed my vision to lead my life in my marriage.

1 Codes:

- ○ consequences of fistula on marital

31:11 My husband divorced me due to this problem. (1445:1487) - D 31: PD-Client 10

My husband divorced me due to this problem.

1 Codes:

- ○ consequences of fistula on marital

31:16 He liked me only as if I would be healthy and live with him but due to (2398:2493) - D 31: PD-Client 10

He liked me only as if I would be healthy and live with him but due to my fistula he avoided me.

1 Codes:

○ consequences of fistula on marital

33:8 married new wife and build a new family, his new wife also left him by (783:955) - D 33: PD-Client 11

married new wife and build a new family, his new wife also left him by telling him that you couldn't support your first wife. Then he came searching for me at health center.

1 Codes:

○ consequences of fistula on marital

○ consequences of fistula on social life

Comment: by toshiba

> *not attending community gatherings frequently*

8 Quotations:

16:8 if the woman faced fistula she can't go market, mosque/church, can't g (1227:1357) - D 16: PD-Client 3

if the woman faced fistula she can't go market, mosque/church, can't go anywhere outside home. She fears to sit with other people.

2 Codes:

○ consequences of fistula on social life / ○ problems due to fistula

16:33 I can't lead my social life going here and, there is health problem li (3623:3738) - D 16: PD-Client 3

I can't lead my social life going here and, there is health problem like bad smile. I can't work my daily activities

1 Codes:

○ consequences of fistula on social life

23:7 the problem I had seen on me was that I did not able to be with neighb (389:462) - D 23: PD-Client 6

the problem I had seen on me was that I did not able to be with neighbors

2 Codes:

○ consequences of fistula on social life / ○ problems due to fistula

24:18 I feel bad when I do not engage in human social issues. For instance, (2061:2255) - D 24: PD-Client 7

I feel bad when I do not engage in human social issues. For instance, I'm not going anywhere like my friends and When a person dies, I don't go to mourn. It's all about sitting alone in my house.

3 Codes:

○ consequences of fistula on social life / ○ internal suffering / ○ life after fistula

24:20 Another difficult part of my problem is separating myself from my neig (2257:2388) - D 24: PD-Client 7

Another difficult part of my problem is separating myself from my neighbors, failed to go to market and usual livelihood activities.

2 Codes:

○ consequences of fistula on social life / ○ life after fistula

24:30 This obstetric fistula impacted my life as it caused my life to be pas (3786:3913) - D 24: PD-Client 7

This obstetric fistula impacted my life as it caused my life to be passed solely without sharing good and bad with my neighbors,

1 Codes:

○ consequences of fistula on social life

25:22 like my friends and neighbors, I can`t go out and actively participate (4137:4292) - D 25: PD-Client 8

like my friends and neighbors, I can`t go out and actively participate in the social issues. For instance, I can't go to market and do everything by myself.

1 Codes:

○ consequences of fistula on social life

31:6 They faced with very difficult problems, for instance women with urine (382:595) - D 31: PD-Client 10

They faced with very difficult problems, for instance women with urine drippings cannot able to work and struggle to survive outside being exposed to surrounding peoples as they ashamed having contact with peoples.

2 Codes:

○ consequences of fistula on daily cativity / ○ consequences of fistula on social life

---

○ coping with difficult social life

Comment: by toshiba

tolerance

14 Quotations:

1:20 you will prefer loneliness not to see any one. (1318:1363) - D 1: PD-Client 1

you will prefer loneliness not to see any one.

1 Codes:

○ coping with difficult social life

1:39 After that, I will send either my husband or my baby to market and to (2690:2788) - D 1: PD-Client 1

After that, I will send either my husband or my baby to market and to “Idir”, ”dogayii”, ”wedding”

2 Codes:

○ coping with difficult social life / ○ coping with social life

1:46 When any one came my home, even though I cooked the food, I order my s (3358:3677) - D 1: PD-Client 1

When any one came my home, even though I cooked the food, I order my servant to gave them and told them had a job inside not to eat with them, not to harm their appetite. Beside me, I will send my servant to drink coffee with neighbors and

” I prefer being inside home for hating their facial expression and gossip.”

1 Codes:

○ coping with difficult social life

2:16 you will prefer lonliness and even to have aseparate home in the fores (1421:1519) - D 2: PD-Client 2

you will prefer lonliness and even to have aseparate home in the forest inorder not to see any one.

1 Codes:

○ coping with difficult social life

2:17 When peole come to your home to reassure you, youwill rather prefer to (1521:1615) - D 2: PD-Client 2

When peole come to your home to reassure you, youwill rather prefer to insult and shout at them

1 Codes:

○ coping with difficult social life

2:21 you will Isolate your self (1781:1806) - D 2: PD-Client 2

you will Isolate your self

1 Codes:

○ coping with difficult social life

2:23 better to die(performing suicide) (1845:1879) - D 2: PD-Client 2

better to die(performing suicide)

2 Codes:

○ coping with difficult social life / ○ response to fistula problems

2:39 even I had decided to live in the forest like wild animal in order not (3889:3983) - D 2: PD-Client 2

even I had decided to live in the forest like wild animal in order not to look and hear any one

1 Codes:

○ coping with difficult social life

2:42 I had also isolated my self (e.g. wedding, lekso, Ikub). (4216:4272) - D 2: PD-Client 2

I had also isolated my self (e.g. wedding, lekso, Ikub).

1 Codes:

○ coping with difficult social life

16:9 I myself didn’t leave home when I was at home before coming here. (1359:1423) - D 16: PD-Client 3

I myself didn’t leave home when I was at home before coming here.

1 Codes:

○ coping with difficult social life

23:8 always I stay home alone. (466:491) - D 23: PD-Client 6

always I stay home alone.

1 Codes:

○ coping with difficult social life

24:13 Still no one knows about this case except my mom. I hide all my neighb (1004:1200) - D 24: PD-Client 7

Still no one knows about this case except my mom. I hide all my neighbors and even some of my children about my fistula case. Almost always, my neighbors or other individuals greet me at the door.

1 Codes:

○ coping with difficult social life

25:15 I didn`t disclose the case to my neighbors. They didn`t know the situa (2113:2776) - D 25: PD-Client 8

I didn`t disclose the case to my neighbors. They didn`t know the situation I am in, because I keep my hygiene every day by washing my clothes and taking a shower. No one knows about the challenge I am facing and privacy. Some times to hide my fistula problem, I restrict myself from drinking water. So, I get some relief from dribbling of urine, bad odor and fear of individuals perception. Even by doing this, I am not confident to closely sit and play with my neighbors and friends as well as I am not going my neighbor's house. I never told to anybody since the fistula is shameful. I hide it not to be stigmatized by my neighbors and others due to my fistula.

3 Codes:

○ coping with difficult social life / ○ coping with odours / ○ coping with wetness

25:30 Due to this result she left her usual living home in rural area and de (5007:5139) - D 25: PD-Client 8

Due to this result she left her usual living home in rural area and decided to lead life grow her 3 children in Urban rental house.

3 Codes:

○ coping with difficult social life / ○ coping with odours / ○ coping with wetness

---

○ coping with fistula challenges

Comment: by toshiba

with praying

0 Quotations

○ coping with marital responsibilities

Comment: by toshiba

> *allowing husband to marry another wife*

0 Quotations

○ coping with odours

Comment: by toshiba

> *by washing clothes*

10 Quotations:

1:11 you should have to harry to clean up the area (787:831) - D 1: PD-Client 1

you should have to harry to clean up the area

1 Codes:

○ coping with odours

1:14 when you go out of your home you should have to at least wear two-thre (877:983) - D 1: PD-Client 1

when you go out of your home you should have to at least wear two-three clothes to hide the wetness & odor

2 Codes:

○ coping with odours / ○ coping with wetness

20:20 . I put rubber sheet, when I sleep and wear pad to keep the urine but (2651:2787) - D 20: PD-Client 5

. I put rubber sheet, when I sleep and wear pad to keep the urine but washing/cleaning those materials and my clothing was very difficult

2 Codes:

○ coping with odours / ○ coping with wetness

21:35 Its only to reduce urine. You can see our ward you can’t imagine how (6974:7142) - D 21: PD-BSc Nurse

Its only to reduce urine. You can see our ward you can’t imagine how it is free from smelling this much this is because we advised them to take adequate fluid and water

1 Codes:

○ coping with odours

24:24 I my self-decided to be alone in my home due to fear of bad smell my f (2826:2909) - D 24: PD-Client 7

I my self-decided to be alone in my home due to fear of bad smell my fistula cause.

2 Codes:

○ coping with odours / ○ problems due to fistula

24:29 I have been using pad of clothes to my perineum to trap the leaking ur (3531:3784) - D 24: PD-Client 7

I have been using pad of clothes to my perineum to trap the leaking urine to overcome my fistula cases. In addition, I have been frequently changing her clothes, and I stay in My separated room to stay away from attention of peoples who come to my house.

2 Codes:

○ coping with odours / ○ coping with wetness

25:15 I didn`t disclose the case to my neighbors. They didn`t know the situa (2113:2776) - D 25: PD-Client 8

I didn`t disclose the case to my neighbors. They didn`t know the situation I am in, because I keep my hygiene every day by washing my clothes and taking a shower. No one knows about the challenge I am facing and privacy. Some times to hide my fistula problem, I restrict myself from drinking water. So, I get some relief from dribbling of urine, bad odor and fear of individuals perception. Even by doing this, I am not confident to closely sit and play with my neighbors and friends as well as I am not going my neighbor's house. I never told to anybody since the fistula is shameful. I hide it not to be stigmatized by my neighbors and others due to my fistula.

3 Codes:

○ coping with difficult social life / ○ coping with odours / ○ coping with wetness

25:30 Due to this result she left her usual living home in rural area and de (5007:5139) - D 25: PD-Client 8

Due to this result she left her usual living home in rural area and decided to lead life grow her 3 children in Urban rental house.

3 Codes:

○ coping with difficult social life / ○ coping with odours / ○ coping with wetness

25:33 To overcome this challenge, I abstain myself from drinking water.so th (5622:5989) - D 25: PD-Client 8

To overcome this challenge, I abstain myself from drinking water.so that the dribbling of urine become decrease. On top this, there is a traditional medication called "qoricha michii"(Damaakasee), with this medication first I boil a water then add this medication into the boiled water then I sit on it for some minutes, finally I wash my vagina with this cool water.

2 Codes:

○ coping with odours / ○ coping with wetness

27:13 I had separated myself from social activities such as 'equib' and 'idi (888:1069) - D 27: PD-Client 9

I had separated myself from social activities such as 'equib' and 'idir' due to fear of bad odor resulted from fistula by myself but openly no one had insulted and discriminated me.

1 Codes:

○ coping with odours

○ coping with social life

Comment: by toshiba

> *not attending public meetings*

5 Quotations:

1:21 When people come to your home to reassure you, you will rather prefer (1365:1539) - D 1: PD-Client 1

When people come to your home to reassure you, you will rather prefer to insult and shout at them and consider as they came to see your smells and share your secret to others.

2 Codes:

○ coping with social life / ○ response to social life

1:27 makes you to isolate yourself in all direction. (1699:1745) - D 1: PD-Client 1

makes you to isolate yourself in all direction.

1 Codes:

○ coping with social life

1:32 my husband and my parents tries to hide my secret and game support (2088:2153) - D 1: PD-Client 1

my husband and my parents tries to hide my secret and game support

1 Codes:

○ coping with social life

1:39 After that, I will send either my husband or my baby to market and to (2690:2788) - D 1: PD-Client 1

After that, I will send either my husband or my baby to market and to "Idir", "dogayii", "wedding"

2 Codes:

○ coping with difficult social life / ○ coping with social life

31:18 but after cleaning my body and close I can attend mourning places. (2554:2620) - D 31: PD-Client 10

but after cleaning my body and close I can attend mourning places.

1 Codes:

○ coping with social life

○ coping with stigma and discrimination

Comment: by toshiba

> *not went out of home*

0 Quotations

○ coping with wetness

Comment: by toshiba

> *escaping self*

10 Quotations:

1:13 when you go out of your home you should have to at least wear two-thre (877:975) - D 1: PD-Client 1

when you go out of your home you should have to at least wear two-three clothes to hide the wetness

1 Codes:

○ coping with wetness

1:14 when you go out of your home you should have to at least wear two-thre (877:983) - D 1: PD-Client 1

when you go out of your home you should have to at least wear two-three clothes to hide the wetness & odor

2 Codes:

○ coping with odours / ○ coping with wetness

20:20 . I put rubber sheet, when I sleep and wear pad to keep the urine but (2651:2787) - D 20: PD-Client 5

. I put rubber sheet, when I sleep and wear pad to keep the urine but washing/cleaning those materials and my clothing was very difficult

2 Codes:

○ coping with odours / ○ coping with wetness

21:30 Second Mostly fistula patients take few or not take water/fluid for th (6092:6248) - D 21: PD-BSc Nurse

Second Mostly fistula patients take few or not take water/fluid for the purpose of minimizing the amount of urine. It’s true it minimizes the amount of urine

1 Codes:

○ coping with wetness

24:29 I have been using pad of clothes to my perineum to trap the leaking ur (3531:3784) - D 24: PD-Client 7

I have been using pad of clothes to my perineum to trap the leaking urine to overcome my fistula cases. In addition, I have been frequently changing her clothes, and I stay in My separated room to stay away from attention of peoples who come to my house.

2 Codes:

○ coping with odours / ○ coping with wetness

25:15 I didn`t disclose the case to my neighbors. They didn`t know the situa (2113:2776) - D 25: PD-Client 8

I didn`t disclose the case to my neighbors. They didn`t know the situation I am in, because I keep my hygiene every day by washing my clothes and taking a shower. No one knows about the challenge I am facing and privacy. Some times to hide my fistula problem, I restrict myself from drinking water. So, I get some relief from dribbling of urine, bad odor and fear of individuals perception. Even by doing this, I am not confident to closely sit and play with my neighbors and friends as well as I am not going my neighbor’s house. I never told to anybody since the fistula is shameful. I hide it not to be stigmatized by my neighbors and others due to my fistula.

3 Codes:

○ coping with difficult social life / ○ coping with odours / ○ coping with wetness

25:30 Due to this result she left her usual living home in rural area and de (5007:5139) - D 25: PD-Client 8

Due to this result she left her usual living home in rural area and decided to lead life grow her 3 children in Urban rental house.

3 Codes:

○ coping with difficult social life / ○ coping with odours / ○ coping with wetness

25:33 To overcome this challenge, I abstain myself from drinking water.so th (5622:5989) - D 25: PD-Client 8

To overcome this challenge, I abstain myself from drinking water.so that the dribbling of urine become decrease. On top this, there is a traditional medication called "qoricha michii"(Damaakasee), with this medication first I boil a water then add this medication into the boiled water then I sit on it for some minutes, finally I wash my vagina with this cool water.

2 Codes:

○ coping with odours / ○ coping with wetness

27:14 To cope up with my obstetric fistula, I have been using modus and fre (1071:1319) - D 27: PD-Client 9

To cope up with my obstetric fistula, I have been using modus and frequently change my clothes. To gain money to buy modus, I ask my family members to give me money by telling them as I have abdominal pain and as I want to seek health care for it.

1 Codes:

○ coping with wetness

31:9 Those women who had many ladies are a fortunate as they help them in k (813:1122) - D 31: PD-Client 10

Those women who had many ladies are a fortunate as they help them in keeping their cleanliness by washing their closes and the like. For instance now, how Iam surviving with this problem is that, I have many ladies who wash my closes from time to time and make me kept clean and interact and live with peoples.

1 Codes:

○ coping with wetness

○ difficult social life

Comment: by toshiba

> *difficulty of walking with people, attending community gatherings, market places, religious places*

7 Quotations:

1:17 Participating in-social life is painful for us (1097:1144) - D 1: PD-Client 1

Participating in-social life is painful for us

3 Codes:

○ consequences of fistula / ○ difficult social life / ○ painful social life

1:19 Participating in-social life is painful for us since we think of our o (1098:1312) - D 1: PD-Client 1

Participating in-social life is painful for us since we think of our odor always. For example (going to church, “mana dogayii”and “mana ciidha”, even coming to salon to drink coffee with your neighbors is difficult

1 Codes:

○ difficult social life

1:33 the difficulty was socializing as before (e.g. drinking coffee, going (2159:2265) - D 1: PD-Client 1

the difficulty was socializing as before (e.g. drinking coffee, going to market, church, meeting and etc.).

1 Codes:

○ difficult social life

1:36 When I go to church, people will prefer talking about you rather than (2425:2548) - D 1: PD-Client 1

When I go to church, people will prefer talking about you rather than hearing the paster and that will be shocking for you.

4 Codes:

○ difficult social life / ○ internal suffering / ○ painful social life / ○ percieved stigma

1:37 I have a bad memory in the market; “one of the women returned what her (2555:2689) - D 1: PD-Client 1

I have a bad memory in the market; “one of the women returned what her child bought from me after hearing that the material was mine.”

1 Codes:

○ difficult social life

1:41 even the kebele workers will not tell us as there is a meeting and we (2875:2961) - D 1: PD-Client 1

even the kebele workers will not tell us as there is a meeting and we can’t participate

1 Codes:

○ difficult social life

1:55 Oh! It is painful! Painful! All the eyes of people look at you, all t (4866:5070) - D 1: PD-Client 1

Oh! It is painful! Painful! All the eyes of people look at you, all their nose smells your urine and all their ear and mouth talk and hear about you even though you are a single woman living in your home.

4 Codes:

○ difficult social life / ○ internal suffering / ○ painful social life / ○ percieved stigma

---

○ discrimination by community

Comment: by toshiba

> *gossiped by community at market*

6 Quotations:

1:38 “one of the women returned what her child bought from me after hearing (2591:2689) - D 1: PD-Client 1

“one of the women returned what her child bought from me after hearing that the material was mine.”

1 Codes:

○ discrimination by community

2:30 It still continues: our society (community) is the first to dicriminat (2778:3202) - D 2: PD-Client 2

It still continues: our society (community) is the first to dicriminate us and when I pass near bythem on the road they insult me by “Ashmur; shintam lam” as they are saying for cows and even if my cloth is dry,since they know me I am avictim,they will cover their

nose,expel their saliva, turn their face, not want to move with me like to market area, not inform me when they gofor social (e.g.” Lekso bet injera mewsed ”).

1 Codes:

○ discrimination by community

2:32 the kebele workers will not tell us as there is ameeting and we can’t (3314:3394) - D 2: PD-Client 2

the kebele workers will not tell us as there is ameeting and we can’t participate

1 Codes:

○ discrimination by community

2:41 When I go to market after wearing my pad, when they see the wetted clo (4048:4215) - D 2: PD-Client 2

When I go to market after wearing my pad, when they see the wetted clothe, they say ” ichi nat ichi nat iyalu yayugnal” after that, I will go back to home with crying.

1 Codes:

○ discrimination by community

21:34 unwanted actions such as covering nose and mouth, sitting far from her (6559:6668) - D 21: PD-BSc Nurse

unwanted actions such as covering nose and mouth, sitting far from her and other actions that torch her mind.

2 Codes:

○ discrimination by community / ○ discrimination by family

25:16 Before I came to my current resident, I was living with my father in a (2779:3118) - D 25: PD-Client 8

Before I came to my current resident, I was living with my father in a very remote area. At that time, my father and other family members also know my case. I was stigmatized and discriminated by my family (father, wife of my father and sister in law) and, intimate neighbors due to bad odor caused by continuous leakage of feces and urine.

2 Codes:

○ discrimination by community / ○ discrimination due to fistula

---

○ discrimination by family

Comment: by toshiba

> *family had a bad ffeeling for her*

3 Quotations:

21:34 unwanted actions such as covering nose and mouth, sitting far from her (6559:6668) - D 21: PD-BSc Nurse

unwanted actions such as covering nose and mouth, sitting far from her and other actions that torch her mind.

2 Codes:

○ discrimination by community / ○ discrimination by family

33:10 His family encouraged and advised him to marry other wife telling him (1088:1197) - D 33: PD-Client 11

His family encouraged and advised him to marry other wife telling him as Iam not able to bear a child for him.

3 Codes:

○ discrimination by family / ○ discrimination by neigbors / ○ effects of fistula on sexual reproductive health

33:11 After I developed this problem I faced nothing from my parents and my (1374:1573) - D 33: PD-Client 11

After I developed this problem I faced nothing from my parents and my community except from my husband. He liked me only as if I would be healthy and live with him but due to my fistula he avoided me.

1 Codes:

○ discrimination by family

○ discrimination by neigbors

Comment: by toshiba

> *refused to eat with her*

2 Quotations:

25:29 Few of my intimate neighbor was stigmatized and discriminated me due t (4849:5006) - D 25: PD-Client 8

Few of my intimate neighbor was stigmatized and discriminated me due to bad odor caused by continuous leakage of feces and urine. They refused to eat with me.

1 Codes:

○ discrimination by neigbors

33:10 His family encouraged and advised him to marry other wife telling him (1088:1197) - D 33: PD-Client 11

His family encouraged and advised him to marry other wife telling him as Iam not able to bear a child for him.

3 Codes:

○ discrimination by family / ○ discrimination by neigbors / ○ effects of fistula on sexual reproductive health

---

○ discrimination due to fistula

Comment: by toshiba

> *removed from all community and family participations*

6 Quotations:

2:46 But,the big problem was people were not buying materials from me : not (4607:4695) - D 2: PD-Client 2

But,the big problem was people were not buying materials from me : not drunk the "arake"

2 Codes:

○ discrimination due to fistula / ○ problems due to fistula

18:32 My family refuse to accept me as a functional person after this diseas (3727:4157) - D 18: PD-Client 4

My family refuse to accept me as a functional person after this disease. Totally they removed me from any community and family participation. They like a woman who has money, lives in good relationship with her husband, and have and care children. In our community people work and live together, they sell and buy in group, they care children together and eat together. After I face this fistula, I am out of this all activities.

3 Codes:

○ discrimination due to fistula / ○ problems due to fistula / ○ problems experienced after first birth

18:33 No, Oromo do everything together! In Arsi culture people live together (4254:4487) - D 18: PD-Client 4

No, Oromo do everything together! In Arsi culture people live together, celebrate (chidha), Dewa and other different ceremony together. But a woman with this problem cannot be seen as a woman, they consider you as you are out of mind!

1 Codes:

○ discrimination due to fistula

20:16 When I go to wedding ceremony and other social activities people try t (2013:2148) - D 20: PD-Client 5

When I go to wedding ceremony and other social activities people try to stay far from me or cover their nose and mouth and talk about me

1 Codes:

○ discrimination due to fistula

20:19 Sometimes friends came to my house to ask me but they stay far from me (2492:2650) - D 20: PD-Client 5

Sometimes friends came to my house to ask me but they stay far from me. when I go to wedding or condolence people cover, their nose and try to stay far from me

1 Codes:

○ discrimination due to fistula

25:16 Before I came to my current resident, I was living with my father in a (2779:3118) - D 25: PD-Client 8

Before I came to my current resident, I was living with my father in a very remote area. At that time, my father and other family members also know my case. I was stigmatized and discriminated by my family (father, wife of my father and sister in law) and, intimate neighbors due to bad odor caused by continuous leakage of feces and urine.

2 Codes:

○ discrimination by community / ○ discrimination due to fistula

---

○ divorced marriage

Comment: by toshiba

> *not with husband*

1 Quotations:

18:4 Divorced/separated (396:414) - D 18: PD-Client 4

Divorced/separated

2 Codes:

○ divorced marriage / ○ marital status

○ effects of fistula on economy

Comment: by toshiba

lived with children without any support from husband, not able to participate in productive activities

2 Quotations:

31:13 He left to me all our children and lived himself alone with his new wi (1869:1970) - D 31: PD-Client 10

He left to me all our children and lived himself alone with his new wife using my properties with her.

1 Codes:

○ effects of fistula on economy

31:14 Me and my children lost support from him (1972:2011) - D 31: PD-Client 10

Me and my children lost support from him

1 Codes:

○ effects of fistula on economy

○ effects of fistula on routine activity

Comment: by toshiba

can't performe routine activities

1 Quotations:

27:10 I missed love from my husband; (584:614) - D 27: PD-Client 9

I missed love from my husband;

1 Codes:

○ effects of fistula on routine activity

○ effects of fistula on sexual reproductive health

Comment: by toshiba

lost her marriage, insulted, difficult srh

9 Quotations:

1:59 Fistula made you to be full of fear when you think of sexual life and (5453:5635) - D 1: PD-Client 1

Fistula made you to be full of fear when you think of sexual life and it makes you different from other women (e.g. vaginal secretion with urine) which decrease your feeling for sex.

2 Codes:

○ consequences of fistula / ○ effects of fistula on sexual reproductive health

2:53 The big problem was, when you thought about sexual intercourse,having (5590:5817) - D 2: PD-Client 2

The big problem was, when you thought about sexual intercourse,having marriage,having baby; this is to say,you will loose your confidence of talking with male(boy) , the impact will make you to loose your desire and satisfaction

3 Codes:

○ consequences of fistula / ○ effects of fistula on sexual reproductive health / ○ problems due to fistula

16:34 no sexual life at all. (3741:3764) - D 16: PD-Client 3

no sexual life at all.

1 Codes:

○ effects of fistula on sexual reproductive health

20:15 We didn’t have much relationship(like sleeping together) after once I (1872:1960) - D 20: PD-Client 5

We didn’t have much relationship(like sleeping together) after once I developed fistula.

1 Codes:

○ effects of fistula on sexual reproductive health

23:17 She explained the impact of fistula on her live as it caused her to fa (1251:1347) - D 23: PD-Client 6

She explained the impact of fistula on her live as it caused her to fail to gain her sexual need.

1 Codes:

○ effects of fistula on sexual reproductive health

24:32 Furthermore, my fistula impacts my sexual life, I complain that i have (4005:4123) - D 24: PD-Client 7

Furthermore, my fistula impacts my sexual life, I complain that i haven’t had sexual intercourse since I faced fistula.

1 Codes:

○ effects of fistula on sexual reproductive health

27:16 In the same manner I failed to fulfill my sexual need. (1439:1494) - D 27: PD-Client 9

In the same manner I failed to fulfill my sexual need.

1 Codes:

○ effects of fistula on sexual reproductive health

33:7 my husband said she cannot able to have children (730:777) - D 33: PD-Client 11

my husband said she cannot able to have children

1 Codes:

○ effects of fistula on sexual reproductive health

33:10 His family encouraged and advised him to marry other wife telling him (1088:1197) - D 33: PD-Client 11

His family encouraged and advised him to marry other wife telling him as Iam not able to bear a child for him.

3 Codes:

○ discrimination by family / ○ discrimination by neigbors / ○ effects of fistula on sexual reproductive health

---

○ for which baby got fistula

Comment: by toshiba

> *acquired fistula on the first child*

1 Quotations:

1:31 and got this problem on my third baby (2049:2085) - D 1: PD-Client 1

and got this problem on my third baby

2 Codes:

○ for which baby got fistula / ○ time when got fistula

○ internal suffering

Comment: by toshiba

> *most of the time felt bad and loneliness*

12 Quotations:

1:23 consider as they came to see your smells and share your secret to othe (1467:1539) - D 1: PD-Client 1

consider as they came to see your smells and share your secret to others.

2 Codes:

○ internal suffering / ○ percieved stigma

1:24 Your suffering is also from inside (1548:1581) - D 1: PD-Client 1

Your suffering is also from inside

2 Codes:

○ internal suffering / ○ percieved stigma

1:26 extreme suspiciousness (1676:1697) - D 1: PD-Client 1

extreme suspiciousness

2 Codes:

○ internal suffering / ○ percieved stigma

1:28 even if no-one knows about your problem, your extreme suspiciousness m (1630:1745) - D 1: PD-Client 1

even if no-one knows about your problem, your extreme suspiciousness makes you to isolate yourself in all direction.

2 Codes:

○ internal suffering / ○ percieved stigma

1:36 When I go to church, people will prefer talking about you rather than (2425:2548) - D 1: PD-Client 1

When I go to church, people will prefer talking about you rather than hearing the paster and that will be shocking for you.

4 Codes:

○ difficult social life / ○ internal suffering / ○ painful social life / ○ percieved stigma

1:55 Oh! It is painful! Painful! All the eyes of people look at you, all t (4866:5070) - D 1: PD-Client 1

Oh! It is painful! Painful! All the eyes of people look at you, all their nose smells your urine and all their ear and mouth talk and hear about you even though you are a single woman living in your home.

4 Codes:

○ difficult social life / ○ internal suffering / ○ painful social life / ○ percieved stigma

2:14 you will be full of suspicion that,even if they talk about any thing n (1042:1210) - D 2: PD-Client 2

you will be full of suspicion that,even if they talk about any thing nad lough ,you will consider as they are talking about you eventhough they may not know about you.

2 Codes:

○ internal suffering / ○ percieved stigma

2:43 Even, I was afraid of even participating in community meetings and c (4280:4382) - D 2: PD-Client 2

Even, I was afraid of even participating in community meetings and celebrations(e.g. kebele meeting)

2 Codes:

○ internal suffering / ○ percieved stigma

20:17 These makes me to feel very bad and think why I am the only person tro (2151:2292) - D 20: PD-Client 5

These makes me to feel very bad and think why I am the only person troubling with this problem and why it lucks medical treatment and so forth

2 Codes:

○ internal suffering / ○ psychological problems due to fistula

24:18 I feel bad when I do not engage in human social issues. For instance, (2061:2255) - D 24: PD-Client 7

I feel bad when I do not engage in human social issues. For instance, I'm not going anywhere like my friends and When a person dies, I don't go to mourn. It's all about sitting alone in my house.

3 Codes:

○ consequences of fistula on social life / ○ internal suffering / ○ life after fistula

24:31 this provoke my feelings of sadness, depression and lead me to cry mos (3919:4002) - D 24: PD-Client 7

this provoke my feelings of sadness, depression and lead me to cry most of the time.

2 Codes:

○ internal suffering / ○ psychological problems due to fistula

27:11 I have continuous mental stress about my own health and the future lif (615:700) - D 27: PD-Client 9

I have continuous mental stress about my own health and the future life of my child.

2 Codes:

○ internal suffering / ○ psychological problems due to fistula

---

○ life after fistula

Comment: by toshiba

> *can't lead life as before*

2 Quotations:

24:18 I feel bad when I do not engage in human social issues. For instance, (2061:2255) - D 24: PD-Client 7

I feel bad when I do not engage in human social issues. For instance, I'm not going anywhere like my friends and When a person dies, I don't go to mourn. It's all about sitting alone in my house.

3 Codes:

○ consequences of fistula on social life / ○ internal suffering / ○ life after fistula

24:20 Another difficult part of my problem is separating myself from my neig (2257:2388) - D 24: PD-Client 7

Another difficult part of my problem is separating myself from my neighbors, failed to go to market and usual livelihood activities.

2 Codes:

○ consequences of fistula on social life / ○ life after fistula

---

○ life after repair

Comment: by toshiba

> *very good progress*

3 Quotations:

2:55 But, after surgery, glory to God that, I have married and even have tw (5899:6004) - D 2: PD-Client 2

But, after surgery, glory to God that, I have married and even have two children and leading a happy life.

1 Codes:

○ life after repair

2:57 When you go to the society after repair, the people didn't accept as i (6168:6248) - D 2: PD-Client 2

When you go to the society after repair, the people didn't accept as it is true

1 Codes:

○ life after repair

2:60 I started to have avision, talk and go with people and have ahope. But (6540:6707) - D 2: PD-Client 2

I started to have avision, talk and go with people and have ahope. But still when I got angry, I am easy to cry,to shout and irritable eventhough the degree is small.

1 Codes:

○ life after repair

○ lived with fistula with no treatment

Comment: by toshiba

> *for twenty to thirty years*

1 Quotations:

1:8 I have stayed with this disease for fifteen (15) years without getting (400:492) - D 1: PD-Client 1

I have stayed with this disease for fifteen (15) years without getting any medical treatment.

1 Codes:

○ lived with fistula with no treatment

○ lived with whom

Comment: by toshiba

> *lived with family while in problem*

3 Quotations:

18:35 All these years I lived alone. But last time I left my house and gone (4670:4774) - D 18: PD-Client 4

All these years I lived alone. But last time I left my house and gone a place even I don't know(metifat).

2 Codes:

○ lived with whom / ○ lived with whom after fistula

24:19 My children are with me and they understand me also. (1793:1844) - D 24: PD-Client 7

My children are with me and they understand me also.

2 Codes:

○ lived with whom / ○ lived with whom after fistula

27:12 And even I have been living with them in the same room. (833:887) - D 27: PD-Client 9

And even I have been living with them in the same room.

2 Codes:

○ lived with whom / ○ lived with whom after fistula

○ lived with whom after fistula

Comment: by toshiba

with family

3 Quotations:

18:35 All these years I lived alone. But last time I left my house and gone (4670:4774) - D 18: PD-Client 4

All these years I lived alone. But last time I left my house and gone a place even I don't know(metifat).

2 Codes:

○ lived with whom / ○ lived with whom after fistula

24:19 My children are with me and they understand me also. (1793:1844) - D 24: PD-Client 7

My children are with me and they understand me also.

2 Codes:

○ lived with whom / ○ lived with whom after fistula

27:12 And even I have been living with them in the same room. (833:887) - D 27: PD-Client 9

And even I have been living with them in the same room.

2 Codes:

○ lived with whom / ○ lived with whom after fistula

○ lived with whom before fistula

Comment: by toshiba

> *with her husband*

0 Quotations

○ living conditions before fistula

Comment: by toshiba

> *lived in rural from low economic family*

1 Quotations:

16:14 I was farmer before the occurrence of problem. I am living in rural ar (1747:1884) - D 16: PD-Client 3

I was farmer before the occurrence of problem. I am living in rural area where difficult to get ambulance. I am from low economic family.

1 Codes:

○ living conditions before fistula

○ marital status

Comment: by toshiba

> *early marriage through family enforcement*

12 Quotations:

1:3 widowed (197:203) - D 1: PD-Client 1

widowed

1 Codes:

○ marital status

2:3 married (193:200) - D 2: PD-Client 2

married

1 Codes:

○ marital status

16:4 Married (443:449) - D 16: PD-Client 3

Married

1 Codes:

○ marital status

18:4 Divorced/separated (396:414) - D 18: PD-Client 4

Divorced/separated

2 Codes:

○ divorced marriage / ○ marital status

20:4 Married (358:365) - D 20: PD-Client 5

Married

1 Codes:

○ marital status

23:3 married (100:106) - D 23: PD-Client 6

married

1 Codes:

○ marital status

24:4 married (132:138) - D 24: PD-Client 7

married

1 Codes:

○ marital status

25:4 widowed (134:140) - D 25: PD-Client 8

widowed

1 Codes:

○ marital status

25:6 can’t read and write (188:207) - D 25: PD-Client 8

can’t read and write

1 Codes:

○ marital status

27:4 separated (152:160) - D 27: PD-Client 9

separated

1 Codes:

○ marital status

31:3 Divorced (137:144) - D 31: PD-Client 10

Divorced

1 Codes:

○ marital status

33:3 Divorced (137:144) - D 33: PD-Client 11

Divorced

1 Codes:

○ marital status

○ marital status of supplementars

2 Quotations:

5:5 married (208:215) - D 5: PD-Husband of the patient

married

1 Codes:

○ marital status of supplementars

17:5 Married (460:466) - D 17: PD-Health officer

Married

1 Codes:

○ marital status of supplementars

○ occupation of supplementars

2 Quotations:

5:6 governmental/teacher (229:248) - D 5: PD-Husband of the patient

governmental/teacher

1 Codes:

○ occupation of supplementars

17:6 Non-Government employee (550:572) - D 17: PD-Health officer

Non-Government employee

1 Codes:

○ occupation of supplementars

○ outcome of first repair

Comment: by toshiba

> *was not healed after first repair, leakage increased, controlled urine, there is urgency*

9 Quotations:

2:70 but no change (8089:8102) - D 2: PD-Client 2

but no change

1 Codes:

○ outcome of first repair

2:72 still no change and he said,she will die soon. (8161:8206) - D 2: PD-Client 2

still no change and he said,she will die soon.

1 Codes:

○ outcome of first repair

17:27 The success is good but it depends on degree and position of fistula. (4555:4905) - D 17: PD-Health officer

The success is good but it depends on degree and position of fistula. If fistula occurred around urethra neck and cervix it is difficult to repair and also the success may be under

question. Sometimes may need repeated surgery. If fistula is at mid vagina it easily cures by single surgery. Sometimes even after repeated surgery there is no success.

1 Codes:

○ outcome of first repair

18:39 I got treatment, surgery was done, the amount of urine leak decreased (5670:5766) - D 18: PD-Client 4

I got treatment, surgery was done, the amount of urine leak decreased but fail to stope totally.

1 Codes:

○ outcome of first repair

21:24 Yes, there is a chance but its based on the size and site of fistula. (4847:4916) - D 21: PD-BSc Nurse

Yes, there is a chance but its based on the size and site of fistula.

1 Codes:

○ outcome of first repair

25:37 was not corrected and I came back again. (6803:6843) - D 25: PD-Client 8

was not corrected and I came back again.

1 Codes:

○ outcome of first repair

25:43 After surgery, I saw very slight improvement, but the condition was no (7762:7843) - D 25: PD-Client 8

After surgery, I saw very slight improvement, but the condition was not promising.

1 Codes:

○ outcome of first repair

31:21 the treatment makes stop the drippings of my urine. (3396:3447) - D 31: PD-Client 10

the treatment makes stop the drippings of my urine.

1 Codes:

○ outcome of first repair

31:32 I gave birth through operation with dead fetus and developed fistula (5520:5588) - D 31: PD-Client 10

I gave birth through operation with dead fetus and developed fistula

1 Codes:

○ outcome of first repair

○ outcome of living with fistula

1 Quotations:

1:56 The external pressure due to the disease affects also your mentality & (5072:5279) - D 1: PD-Client 1

The external pressure due to the disease affects also your mentality & you start to become pessimist for all even though they were treating you positively and this makes to be aggressive, easy cry and hater.

1 Codes:

○ outcome of living with fistula

○ painful social life

Comment: by toshiba

> *didn't sit and talk with other , not go fro coffee ceremony*

3 Quotations:

1:17 Participating in-social life is painful for us (1097:1144) - D 1: PD-Client 1

Participating in-social life is painful for us

3 Codes:

○ consequences of fistula / ○ difficult social life / ○ painful social life

1:36 When I go to church, people will prefer talking about you rather than (2425:2548) - D 1: PD-Client 1

When I go to church, people will prefer talking about you rather than hearing the paster and that will be shocking for you.

4 Codes:

○ difficult social life / ○ internal suffering / ○ painful social life / ○ percieved stigma

1:55 Oh! It is painful! Painful! All the eyes of people look at you, all t (4866:5070) - D 1: PD-Client 1

Oh! It is painful! Painful! All the eyes of people look at you, all their nose smells your urine and all their ear and mouth talk and hear about you even though you are a single woman living in your home.

4 Codes:

○ difficult social life / ○ internal suffering / ○ painful social life / ○ percieved stigma

---

○ percieved stigma

Comment: by toshiba

fear to join community

24 Quotations:

1:23 consider as they came to see your smells and share your secret to othe (1467:1539) - D 1: PD-Client 1

consider as they came to see your smells and share your secret to others.

2 Codes:

○ internal suffering / ○ percieved stigma

1:24 Your suffering is also from inside (1548:1581) - D 1: PD-Client 1

Your suffering is also from inside

2 Codes:

○ internal suffering / ○ percieved stigma

1:26 extreme suspiciousness (1676:1697) - D 1: PD-Client 1

extreme suspiciousness

2 Codes:

○ internal suffering / ○ percieved stigma

1:28 even if no-one knows about your problem, your extreme suspiciousness m (1630:1745) - D 1: PD-Client 1

even if no-one knows about your problem, your extreme suspiciousness makes you to isolate yourself in all direction.

2 Codes:

○ internal suffering / ○ percieved stigma

1:36 When I go to church, people will prefer talking about you rather than (2425:2548) - D 1: PD-Client 1

When I go to church, people will prefer talking about you rather than hearing the paster and that will be shocking for you.

4 Codes:

○ difficult social life / ○ internal suffering / ○ painful social life / ○ percieved stigma

1:43 over suspicion of being hated (3012:3041) - D 1: PD-Client 1

over suspicion of being hated

1 Codes:

○ percieved stigma

1:47 for hating their facial expression and gossip.” (3630:3677) - D 1: PD-Client 1

for hating their facial expression and gossip.”

2 Codes:

○ percieved stigma / ○ response to social life

1:48 The first discriminator was your mind which will in turn harm your sel (3685:3902) - D 1: PD-Client 1

The first discriminator was your mind which will in turn harm your self-esteem and it goes to neighbors and community. ”wuy!! osoo iji nama nama ajeesa ta’ee ,natu du’aa ture fi funyaan isaan qabatan mataa sidhukkubsa”

1 Codes:

○ percieved stigma

1:55 Oh! It is painful! Painful! All the eyes of people look at you, all t (4866:5070) - D 1: PD-Client 1

Oh! It is painful! Painful! All the eyes of people look at you, all their nose smells your urine and all their ear and mouth talk and hear about you even though you are a single woman living in your home.

4 Codes:

○ difficult social life / ○ internal suffering / ○ painful social life / ○ percieved stigma

2:14 you will be full of suspicion that,even if they talk about any thing n (1042:1210) - D 2: PD-Client 2

you will be full of suspicion that,even if they talk about any thing nad lough ,you will consider as they are talking about you eventhough they may not know about you.

2 Codes:

○ internal suffering / ○ percieved stigma

2:18 consider as they came to see your smells and share your secret to othe (1620:1694) - D 2: PD-Client 2

consider as they came to see your smells and share your secret to others.

1 Codes:

○ percieved stigma

2:20 you will think as all the peoples hate you (1733:1775) - D 2: PD-Client 2

you will think as all the peoples hate you

1 Codes:

○ percieved stigma

2:34 over suspicion of being hated made us to loose a lot (3443:3494) - D 2: PD-Client 2

over suspicion of being hated made us to loose a lot

1 Codes:

○ percieved stigma

2:43 Even, I was afraid of even participating in community meetings and c (4280:4382) - D 2: PD-Client 2

Even, I was afraid of even participating in community meetings and celebrations(e.g. kebele meeting)

2 Codes:

○ internal suffering / ○ percieved stigma

2:54 you will also consider your self as infertile and inferior of all wome (5819:5891) - D 2: PD-Client 2

you will also consider your self as infertile and inferior of all women.

2 Codes:

○ percieved stigma / ○ problems due to fistula

16:22 I didn’t face any discrimination from others but I myself have no inte (2476:2582) - D 16: PD-Client 3

I didn’t face any discrimination from others but I myself have no interest to go and sit with other person.

1 Codes:

○ percieved stigma

18:9 I feel shame to see the community I didn’t gone anywhere like wedding (1058:1192) - D 18: PD-Client 4

I feel shame to see the community I didn’t gone anywhere like wedding ceremony and Regio’s worship because of I feel bad about myself.

1 Codes:

○ percieved stigma

18:17 The problem continued but i decided not to be treated due to fear of d (2033:2106) - D 18: PD-Client 4

The problem continued but i decided not to be treated due to fear of dying

3 Codes:

○ percieved stigma / ○ problems due to fistula / ○ problems experienced after first birth

18:25 At this time, my mind would disturb very much, I feel bad and I prefer (2821:2898) - D 18: PD-Client 4

At this time, my mind would disturb very much, I feel bad and I prefer to die.

2 Codes:

○ percieved stigma / ○ psychological problems due to fistula

18:27 I had no any person who support me I lived alone in one house, I was i (2900:3147) - D 18: PD-Client 4

I had no any person who support me I lived alone in one house, I was in hunger for a long time; I couldn’t buy and sell due to fear of people facial expression. they cover their nose and mouth and starts to talk about me when they look at me.

2 Codes:

○ percieved stigma / ○ problems due to fistula

20:18 They think, the problem is only mine and it was Gods punishment. (2329:2392) - D 20: PD-Client 5

They think, the problem is only mine and it was Gods punishment.

1 Codes:

- ○ percieved stigma

20:21 I myself also think as people talk only about me.so I afraid to see pe (2790:2878) - D 20: PD-Client 5

I myself also think as people talk only about me.so I afraid to see people and stay home.

1 Codes:

- ○ percieved stigma

21:28 Based on what we see here on our patient, stigma is not come mostly fr (5892:5998) - D 21: PD-BSc Nurse

Based on what we see here on our patient, stigma is not come mostly from others its from a women herself.

1 Codes:

- ○ percieved stigma

24:47 hen after I went to local refugee's temporary clinic with my husband b (5660:5943) - D 24: PD-Client 7

hen after I went to local refugee's temporary clinic with my husband but both me and my husband was ashamed to truly tell the inability to control feces and urine and we complained as my problem was prolonged diarrhea that lead me to be mistakenly treated for diarrhea and gone back.

1 Codes:

- ○ percieved stigma

○ physical problems due to fistula

Comment: by toshiba

> *locked leg, inability to walk*

6 Quotations:

18:26 my leg was also locked due to labor, I can't go out even I have diffic (2226:2308) - D 18: PD-Client 4

my leg was also locked due to labor, I can't go out even I have difficulty to stand

1 Codes:

- ○ physical problems due to fistula

20:12 I fail to walk even to stand alone immediately after delivery, the are (1311:1441) - D 20: PD-Client 5

I fail to walk even to stand alone immediately after delivery, the area she cut was very pain full, nothing done to treat the wound

1 Codes:

- physical problems due to fistula

24:11 Starting from the commencement of this problem, I have experienced leg (639:742) - D 24: PD-Client 7

Starting from the commencement of this problem, I have experienced legs pain and difficulty of walking.

1 Codes:

- physical problems due to fistula

24:26 I can`t do it. Leave alone using a toilet, I am incapable of care myse (3150:3240) - D 24: PD-Client 7

I can`t do it. Leave alone using a toilet, I am incapable of care myself. I can`t walk also

1 Codes:

- physical problems due to fistula

25:27 I can’t do excessive physical activity to gain income; I can’t go to m (4681:4756) - D 25: PD-Client 8

I can’t do excessive physical activity to gain income; I can’t go to market

1 Codes:

- physical problems due to fistula

25:31 I couldn’t do more productive activities that would enable me to gain (5140:5277) - D 25: PD-Client 8

I couldn’t do more productive activities that would enable me to gain better in come. This led me to confine my live to be hand to mouth.

1 Codes:

- physical problems due to fistula

○ pressures from husbands new wife

Comment: by toshiba

disliked ,hated and insulted

0 Quotations

○ problem experienced after fisrt repair

1 Quotations:

16:29 After three months I feel when my bladder is full but I can’t control (3055:3130) - D 16: PD-Client 3

After three months I feel when my bladder is full but I can’t control urine.

2 Codes:

○ problem experienced after fisrt repair / ○ problems due to fistula

---

○ problems due to fistula

Comment: by toshiba

difficulty to control fistula

24 Quotations:

2:46 But,the big problem was people were not buying materials from me : not (4607:4695) - D 2: PD-Client 2

But,the big problem was people were not buying materials from me : not drunk the “arake”

2 Codes:

○ discrimination due to fistula / ○ problems due to fistula

2:52 I haven’t seen him after all .Then, when I came back, he married anoth (5473:5581) - D 2: PD-Client 2

I haven’t seen him after all .Then, when I came back, he married another women eventhough we didn’t divorced.

2 Codes:

○ consequences of fistula / ○ problems due to fistula

2:53 The big problem was, when you thought about sexual intercourse,having (5590:5817) - D 2: PD-Client 2

The big problem was, when you thought about sexual intercourse,having marriage,having baby; this is to say,you will loose your confidence of talking with male(boy) , the impact will make you to loose your desire and satisfaction

3 Codes:

○ consequences of fistula / ○ effects of fistula on sexual reproductive health / ○ problems due to fistula

2:54 you will also consider your self as infertile and inferior of all wome (5819:5891) - D 2: PD-Client 2

you will also consider your self as infertile and inferior of all women.

2 Codes:

○ percieved stigma / ○ problems due to fistula

2:76 Since we are irritable and easy to cry to things, (8630:8679) - D 2: PD-Client 2

Since we are irritable and easy to cry to things,

3 Codes:

○ consequences of fistula / ○ problems due to fistula / ○ response to fistula problems

5:15 I have understood the severity of fistula on my Wife (stool& urine inc (1506:1730) - D 5: PD-Husband of the patient

I have understood the severity of fistula on my Wife (stool& urine incontinence) & then tried my best to save her mind and life. In addition to the incontinence, there Was infection on episotomy site full of bleeding and pus

2 Codes:

○ consequences of fistula / ○ problems due to fistula

16:8 if the woman faced fistula she can’t go market, mosque/church, can’t g (1227:1357) - D 16: PD-Client 3

if the woman faced fistula she can’t go market, mosque/church, can’t go anywhere outside home. She fears to sit with other people.

2 Codes:

○ consequences of fistula on social life / ○ problems due to fistula

16:13 My weight was decreased after suffered by this problem. (1605:1661) - D 16: PD-Client 3

My weight was decreased after suffered by this problem.

1 Codes:

○ problems due to fistula

16:29 After three months I feel when my bladder is full but I can’t control (3055:3130) - D 16: PD-Client 3

After three months I feel when my bladder is full but I can’t control urine.

2 Codes:

○ problem experienced after fisrt repair / ○ problems due to fistula

18:15 After delivery urine starts to leak out (1879:1917) - D 18: PD-Client 4

After delivery urine starts to leak out

3 Codes:

○ consequences of fistula / ○ problems due to fistula / ○ problems experienced after first birth

18:17 The problem continued but i decided not to be treated due to fear of d (2033:2106) - D 18: PD-Client 4

The problem continued but i decided not to be treated due to fear of dying

3 Codes:

○ percieved stigma / ○ problems due to fistula / ○ problems experienced after first birth

18:19 Yes, it’s only urine but my leg was also locked due to labor, I can’t (2201:2309) - D 18: PD-Client 4

Yes, it’s only urine but my leg was also locked due to labor, I can’t go out even I have difficulty to stand!

1 Codes:

○ problems due to fistula

18:20 I don’t have much interest for food, I need more water than food. I ha (2342:2483) - D 18: PD-Client 4

I don’t have much interest for food, I need more water than food. I have poor appetite for food and totally I was sick I don’t feel healthy.

1 Codes:

○ problems due to fistula

18:22 I am not strong enough to do anything. (2591:2629) - D 18: PD-Client 4

I am not strong enough to do anything.

1 Codes:

○ problems due to fistula

18:27 I had no any person who support me I lived alone in one house, I was i (2900:3147) - D 18: PD-Client 4

I had no any person who support me I lived alone in one house, I was in hunger for a long time; I couldn’t buy and sell due to fear of people facial expression. they cover their nose and mouth and starts to talk about me when they look at me.

2 Codes:

○ percieved stigma / ○ problems due to fistula

18:32 My family refuse to accept me as a functional person after this diseas (3727:4157) - D 18: PD-Client 4

My family refuse to accept me as a functional person after this disease. Totally they removed me from any community and family participation. They like a woman who has money, lives in good relationship with her husband, and have and care children. In our community people work and live together, they sell and buy in group, they care children together and eat together. After I face this fistula, I am out of this all activities.

3 Codes:

○ discrimination due to fistula / ○ problems due to fistula / ○ problems experienced after first birth

20:13 After few days my urine starts to smell and (michi metagn) I was very (1444:1740) - D 20: PD-Client 5

After few days my urine starts to smell and (michi metagn) I was very sick, I develop fever, head, loss of appetite (I couldn’t eat and drink). and loss of sleep during night. The other difficult problem I faced was when I sit or try to use toilet, I feel heaviness like just something coming out.

2 Codes:

○ problems due to fistula / ○ problems experienced after first birth

21:22 Yes, you are right there are mothers who are discriminated/stigmatize, (4189:4298) - D 21: PD-BSc Nurse

Yes, you are right there are mothers who are discriminated/stigmatize, divorced and different social problems.

1 Codes:

○ problems due to fistula

23:7 the problem I had seen on me was that I did not able to be with neighb (389:462) - D 23: PD-Client 6

the problem I had seen on me was that I did not able to be with neighbors

2 Codes:

○ consequences of fistula on social life / ○ problems due to fistula

24:9 The leaking of urine was started right after I have given child birth, (454:585) - D 24: PD-Client 7

The leaking of urine was started right after I have given child birth, then I faced difficult to control my urine followed by feces.

1 Codes:

○ problems due to fistula

24:24 I my self-decided to be alone in my home due to fear of bad smell my f (2826:2909) - D 24: PD-Client 7

I my self-decided to be alone in my home due to fear of bad smell my fistula cause.

2 Codes:

○ coping with odours / ○ problems due to fistula

25:9 Afterward, I can`t sit with other persons even for a minute. When I wa (727:969) - D 25: PD-Client 8

Afterward, I can`t sit with other persons even for a minute. When I walk the amount urine out flow is not as similar as when I sit. The frequency is minimum on walking, at least I have been experiencing six times urine out flow within an hour.

1 Codes:

○ problems due to fistula

31:7 Women with this problem didn't need life and survival due to offensive (597:749) - D 31: PD-Client 10

Women with this problem didn't need life and survival due to offensive smelling of their urines. Even this smelling is not comfortable for themselves and

1 Codes:

○ problems due to fistula

33:6 I faced with so many problems, (699:728) - D 33: PD-Client 11

I faced with so many problems,

2 Codes:

○ consequences of fistula / ○ problems due to fistula

○ problems experienced after first birth

Comment: by toshiba

urine leakage, incontinence

4 Quotations:

18:15 After delivery urine starts to leak out (1879:1917) - D 18: PD-Client 4

After delivery urine starts to leak out

3 Codes:

○ consequences of fistula / ○ problems due to fistula / ○ problems experienced after first birth

18:17 The problem continued but i decided not to be treated due to fear of d (2033:2106) - D 18: PD-Client 4

The problem continued but i decided not to be treated due to fear of dying

3 Codes:

○ percieved stigma / ○ problems due to fistula / ○ problems experienced after first birth

18:32 My family refuse to accept me as a functional person after this diseas (3727:4157) - D 18: PD-Client 4

My family refuse to accept me as a functional person after this disease. Totally they removed me from any community and family participation. They like a woman who has money, lives in good relationship with her husband, and have and care children. In our community people work and live together, they sell and buy in group, they care children together and eat together. After I face this fistula, I am out of this all activities.

3 Codes:

○ discrimination due to fistula / ○ problems due to fistula / ○ problems experienced after first birth

20:13 After few days my urine starts to smell and (michi metagn) I was very (1444:1740) - D 20: PD-Client 5

After few days my urine starts to smell and (michi metagn) I was very sick, I develop fever, head, loss of appetite (I couldn’t eat and drink). and loss of sleep during night. The other difficult problem I faced was when I sit or try to use toilet, I feel heaviness like just something coming out.

2 Codes:

○ problems due to fistula / ○ problems experienced after first birth

○ psychological problems due to fistula

Comment: by toshiba

mind disturbance, despair ,decide to dei

5 Quotations:

18:25 At this time, my mind would disturb very much, I feel bad and I prefer (2821:2898) - D 18: PD-Client 4

At this time, my mind would disturb very much, I feel bad and I prefer to die.

2 Codes:

○ percieved stigma / ○ psychological problems due to fistula

20:17 These makes me to feel very bad and think why I am the only person tro (2151:2292) - D 20: PD-Client 5

These makes me to feel very bad and think why I am the only person troubling with this problem and why it lucks medical treatment and so forth

2 Codes:

○ internal suffering / ○ psychological problems due to fistula

23:12 The stigma I faced from my husband is that he insulted me by saying ‘x (714:810) - D 23: PD-Client 6

The stigma I faced from my husband is that he insulted me by saying ‘xiroftuu’ or “you bad woman”

1 Codes:

○ psychological problems due to fistula

24:31 this provoke my feelings of sadness, depression and lead me to cry mos (3919:4002) - D 24: PD-Client 7

this provoke my feelings of sadness, depression and lead me to cry most of the time.

2 Codes:

○ internal suffering / ○ psychological problems due to fistula

27:11 I have continuous mental stress about my own health and the future lif (615:700) - D 27: PD-Client 9

I have continuous mental stress about my own health and the future life of my child.

2 Codes:

○ internal suffering / ○ psychological problems due to fistula

---

○ reason for divorce

Comment: by toshiba

> *stayed long time at fistula center*

1 Quotations:

17:19 so that nothing ties to continue their marriage so they divorce easily (3138:3209) - D 17: PD-Health officer

so that nothing ties to continue their marriage so they divorce easily.

1 Codes:

○ reason for divorce

○ reason for not attending social gatherings

Comment: by toshiba

> *fistula and epilepsy*

1 Quotations:

18:51 what I recognize after this disease is everybody likes and respect you (3636:4157) - D 18: PD-Client 4

what I recognize after this disease is everybody likes and respect you if you are healthy. My family refuse to accept me as a functional person after this disease. Totally they removed me from any community and family participation. They like a woman who has money, lives in good relationship with her husband, and have and care children. In our community people work and live together, they sell and buy in group, they care children together and eat together. After I face this fistula, I am out of this all activities.

1 Codes:

○ reason for not attending social gatherings

○ reason for not return to husband home

Comment: by toshiba

> *not cured, family refused, no husband support*

0 Quotations

○ reason for not seek care from facility

Comment: by toshiba

> *not belief what care providers say, no facility nearby, no family support, not get power to decide,no TBA' s communication with patients family*

5 Quotations:

2:67 First, I couldn’t believe in what they say and go back to home by refu (7890:7977) - D 2: PD-Client 2

First, I couldn’t believe in what they say and go back to home by refusing the referral;

1 Codes:

○ reason for not seek care from facility

17:16 even if fistula occurred no body bring her to health facility. (2882:2943) - D 17: PD-Health officer

even if fistula occurred no body bring her to health facility.

2 Codes:

○ reason for not seek care from facility / ○ reason for not seek care from fistula center

20:27 The community thinks as it has no any medication/ treatment. (4167:4227) - D 20: PD-Client 5

The community thinks as it has no any medication/ treatment.

1 Codes:

○ reason for not seek care from facility

20:38 Because of they think it has no any solution or treatment anywhere inc (5429:5582) - D 20: PD-Client 5

Because of they think it has no any solution or treatment anywhere including healthy facility woman’s stay at home with this problem without getting help.

1 Codes:

○ reason for not seek care from facility

27:9 due to the negligence of my husband to take me to health facility (475:540) - D 27: PD-Client 9

due to the negligence of my husband to take me to health facility

1 Codes:

○ reason for not seek care from facility

○ reason for not seek care from fistula center

Comment: by toshiba

> *have no potential, money, and awerness*

1 Quotations:

17:16 even if fistula occurred no body bring her to health facility. (2882:2943) - D 17: PD-Health officer

even if fistula occurred no body bring her to health facility.

2 Codes:

○ reason for not seek care from facility / ○ reason for not seek care from fistula center

---

○ reason for not to return to HF

Comment: by toshiba

> *get paralyzed*

1 Quotations:

18:50 but I refused to go back again due to fear of dying. At that time, I w (5885:6015) - D 18: PD-Client 4

but I refused to go back again due to fear of dying. At that time, I was a child (I was only 15years) I think surgery can kill me.

1 Codes:

○ reason for not to return to HF

○ regular social life

Comment: by toshiba

> *no problem with coffee*

0 Quotations

○ relationships with other fistula cases

0 Quotations

○ residence

17 Quotations:

1:2 rural (174:179) - D 1: PD-Client 1

rural

1 Codes:

○ residence

2:2 urban (172:176) - D 2: PD-Client 2

urban

1 Codes:

○ residence

16:1 Bedele (117:123) - D 16: PD-Client 3

Bedele

1 Codes:

○ residence

16:3 Rural (389:393) - D 16: PD-Client 3

Rural

1 Codes:

○ residence

18:1 Delomena (69:77) - D 18: PD-Client 4

Delomena

1 Codes:

○ residence

18:3 Rural (305:310) - D 18: PD-Client 4

Rural

1 Codes:

- residence

20:1 Dodola (80:86) - D 20: PD-Client 5

Dodola

1 Codes:

- residence

20:3 Rural (302:308) - D 20: PD-Client 5

Rural

1 Codes:

- residence

23:2 Rural (78:82) - D 23: PD-Client 6

Rural

1 Codes:

- residence

24:1 Limu Seka (46:54) - D 24: PD-Client 7

Limu Seka

1 Codes:

- residence

24:3 Rural (110:115) - D 24: PD-Client 7

Rural

1 Codes:

- residence

25:1 Sokoru (49:55) - D 25: PD-Client 8

Sokoru

1 Codes:

- residence

25:3 Urban (111:116) - D 25: PD-Client 8

Urban

1 Codes:

○ residence

27:1 Shabe Sombo (45:56) - D 27: PD-Client 9

Shabe Sombo

1 Codes:

○ residence

27:3 rural (129:134) - D 27: PD-Client 9

rural

1 Codes:

○ residence

31:2 Rural (115:120) - D 31: PD-Client 10

Rural

1 Codes:

○ residence

33:2 Rural (115:120) - D 33: PD-Client 11

Rural

1 Codes:

○ residence

○ residence of supplimentars

4 Quotations:

5:2 sa/nono (24:30) - D 5: PD-Husband of the patient

sa/nono

1 Codes:

○ residence of supplimentars

5:4 urban (187:192) - D 5: PD-Husband of the patient

urban

1 Codes:

○ residence of supplimentars

17:2 Metu town (126:134) - D 17: PD-Health officer

Metu town

1 Codes:

○ residence of supplimentars

17:4 Urban (400:404) - D 17: PD-Health officer

Urban

1 Codes:

○ residence of supplimentars

○ response to enacted stigma

Comment: by toshiba

> *praying, not hearing what said*

0 Quotations

○ response to fistula problems

Comment: by toshiba

> *crying all the day*

3 Quotations:

2:23 better to die(performing suicide) (1845:1879) - D 2: PD-Client 2

better to die(performing suicide)

2 Codes:

○ coping with difficult social life / ○ response to fistula problems

2:76 Since we are irritable and easy to cry to things, (8630:8679) - D 2: PD-Client 2

Since we are irritable and easy to cry to things,

3 Codes:

○ consequences of fistula / ○ problems due to fistula / ○ response to fistula problems

2:80 sometimes you will cry all the day with no reason (8984:9032) - D 2: PD-Client 2

sometimes you will cry all the day with no reason

1 Codes:

○ response to fistula problems

○ response to HCPs advises

Comment: by toshiba

implemented the advise

0 Quotations

○ response to husbands wife insults

0 Quotations

○ response to social life

2 Quotations:

1:21 When people come to your home to reassure you, you will rather prefer (1365:1539) - D 1: PD-Client 1

When people come to your home to reassure you, you will rather prefer to insult and shout at them and consider as they came to see your smells and share your secret to others.

2 Codes:

○ coping with social life / ○ response to social life

1:47 for hating their facial expression and gossip." (3630:3677) - D 1: PD-Client 1

for hating their facial expression and gossip."

2 Codes:

○ percieved stigma / ○ response to social life

○ source of information about fistula

Comment: by toshiba

HCPs

0 Quotations

○ source of information about treatment

Comment: by toshiba

from HEWs, returned fistula cases, radio and community leaders

1 Quotations:

21:2 Those who stay at home get information about the treatment from differ (199:497) - D 21: PD-BSc Nurse

Those who stay at home get information about the treatment from different ways from health extension workers, from patient who treated and back to home place, from radio, community leaders (The zonal health office use community leaders as one of maternal and child health awareness creation method).

1 Codes:

○ source of information about treatment

○ stressed with odours

1 Quotations:

1:18 since we think of our odor always. (1146:1179) - D 1: PD-Client 1

since we think of our odor always.

2 Codes:

○ consequences of fistula / ○ stressed with odours

○ time elapsed to seek care at HF

Comment: by toshiba

one day after delivery

0 Quotations

○ time labour starts

Comment: by toshiba

> *after nine months*

0 Quotations

○ time lived with fistula

0 Quotations

○ time to transfer from govthosp to addis

Comment: by toshiba

> *immediately*

0 Quotations

○ time when fistula reoccure

Comment: by toshiba

> *reoccure during the second delivery at home*

0 Quotations

○ time when got fistula

Comment: by toshiba

> *after delivery of first deceased baby*

7 Quotations:

1:31 and got this problem on my third baby (2049:2085) - D 1: PD-Client 1

and got this problem on my third baby

2 Codes:

○ for which baby got fistula / ○ time when got fistula

2:9 The problem will start when you swallow “injera” and drink water (561:625) - D 2: PD-Client 2

The problem will start when you swallow “injera” and drink water

1 Codes:

○ time when got fistula

2:13 the leakage of urine will increase with your movement (889:943) - D 2: PD-Client 2

the leakage of urine will increase with your movement

1 Codes:

○ time when got fistula

2:37 it starts when they understand the case (3788:3827) - D 2: PD-Client 2

it starts when they understand the case

1 Codes:

○ time when got fistula

2:51 But, after he knows the case he became absent from coming to home (5403:5467) - D 2: PD-Client 2

But, after he knows the case he became absent from coming to home

1 Codes:

○ time when got fistula

24:52 This problem occurred after childbirth. (6453:6494) - D 24: PD-Client 7

This problem occurred after childbirth.

1 Codes:

○ time when got fistula

25:8 As well as, I have encountered the fistula during childbirth. (665:725) - D 25: PD-Client 8

As well as, I have encountered the fistula during childbirth.

1 Codes:

○ time when got fistula

○ times elpased to seek care on foot

0 Quotations

○ tradional care places

Comment: by toshiba

helped and informed to go to fistula traetment center

8 Quotations:

2:68 my families brought me to “Tsebel in the gedam (8040:8085) - D 2: PD-Client 2

my families brought me to “Tsebel in the gedam

1 Codes:

○ tradional care places

17:12 religion leader especially mekana Yesus church and creating awareness (1675:1762) - D 17: PD-Health officer

religion leader especially mekana Yesus church and creating awareness in the community.

1 Codes:

○ tradional care places

17:25 Currently patients do not delay by going to spiritual area like tseba, (3987:4145) - D 17: PD-Health officer

Currently patients do not delay by going to spiritual area like tseba, church…currently the religion leader lead such patients to our center so no such delay.

1 Codes:

○ tradional care places

20:11 traditional birth attendant/a women who was helping me at labor (1013:1075) - D 20: PD-Client 5

traditional birth attendant/a women who was helping me at labor

1 Codes:

○ tradional care places

24:38 I didn`t see any change on my health rather my fistula become more com (4801:4890) - D 24: PD-Client 7

I didn`t see any change on my health rather my fistula become more complicated and worsen.

1 Codes:

- ○ tradional care places

24:44 For myself, I was seen by traditional birth attendant (TBA) at the fir (5420:5497) - D 24: PD-Client 7

For myself, I was seen by traditional birth attendant (TBA) at the first time.

1 Codes:

- ○ tradional care places

33:13 The church encourages me a lot and advised me every day, as I will be (1855:2095) - D 33: PD-Client 11

The church encourages me a lot and advised me every day, as I will be cured, they told me not to worry and disturbed and as they will help me for my fistula problem. There for the church supports me totally and made me to reach to this time.

1 Codes:

- ○ tradional care places

33:18 The church supports me by raising money and advise my husband to bring (3769:3928) - D 33: PD-Client 11

The church supports me by raising money and advise my husband to bring me to fistula treatment center, but he finished those money and refused to bring me there

1 Codes:

- ○ tradional care places

○ traditional call of fistula

1 Quotations:

1:30 Both me and my families didn't know the disease was fistula for at lea (1857:2047) - D 1: PD-Client 1

Both me and my families didn't know the disease was fistula for at least ten years and consider as it happens due to urinating outside home after delivery and traditional call it " michii"

1 Codes:

- ○ traditional call of fistula

○ types of supplementars

Comment: by toshiba

prevention officer

4 Quotations:

5:1 husband of the client (120:142) - D 5: PD-Husband of the patient

husband of the client

1 Codes:

○ types of supplementars

17:1 prevention officer (37:54) - D 17: PD-Health officer

prevention officer

1 Codes:

○ types of supplementars

17:8 Prevention officer. (867:885) - D 17: PD-Health officer

Prevention officer.

1 Codes:

○ types of supplementars

21:5 HEALTH CARE PROVIDER (0:19) - D 21: PD-BSc Nurse

HEALTH CARE PROVIDER

1 Codes:

○ types of supplementars

○ what makes to seek care

Comment: by toshiba

increased urine incontinence

3 Quotations:

1:54 After knowing about this disease and started to meet previous victims (4479:4744) - D 1: PD-Client 1

After knowing about this disease and started to meet previous victims of fistula in my kebele, getting them, discussing with them and staying with them all helped me a lot to forget the disease and made me ready to go for treatment, gave me a hope to live in life.

1 Codes:

○ what makes to seek care

24:59 She was a reason for me to come to Jimma University Medical Center. (7255:7322) - D 24: PD-Client 7

She was a reason for me to come to Jimma University Medical Center.

1 Codes:

○ what makes to seek care

33:17 My fistula problems of urine and fecal incontinence enforced me to go (3631:3767) - D 33: PD-Client 11

My fistula problems of urine and fecal incontinence enforced me to go health center

My families motivates and supported me to go there.

1 Codes:

○ what makes to seek care

○ when got obstetric fistula

Comment: by toshiba

> *during birth of first baby*

0 Quotations
